# A Comprehensive Guide for Performing Sample Preparation and Top-Down Protein Analysis

**DOI:** 10.3390/proteomes5020011

**Published:** 2017-04-07

**Authors:** Matthew P. Padula, Iain J. Berry, Matthew B. O′Rourke, Benjamin B.A. Raymond, Jerran Santos, Steven P. Djordjevic

**Affiliations:** 1 Proteomics Core Facility, University of Technology Sydney, PO Box 123, Broadway, Ultimo NSW 2007, Australia; iain.j.berry@student.uts.edu.au (I.J.B.); matthew.orourke@sydney.edu.au (M.B.O.R.); benjamin.raymond@uts.edu.au (B.B.A.R.); jerran.santos@uts.edu.au (J.S.); steven.djordjevic@uts.edu.au (S.P.D.); 2 Infection, Immunity and Innovation Institute, University of Technology Sydney, PO Box 123, Broadway, Ultimo NSW 2007, Australia; 3 Mass Spectrometry Core Facility, Charles Perkins Centre, University of Sydney, The Hub D17, Sydney, Camperdown NSW 2006, Australia; 4 Advanced Tissue Regeneration & Drug Delivery Group, University of Technology Sydney, PO Box 123, Broadway, Ultimo NSW 2007, Australia

**Keywords:** Proteomics, Top-Down, Bottom-up, Mass spectrometry, Electrophoresis, Isoelectric focusing, Proteoform, Chromatography

## Abstract

Methodologies for the global analysis of proteins in a sample, or proteome analysis, have been available since 1975 when Patrick O′Farrell published the first paper describing two-dimensional gel electrophoresis (2D-PAGE). This technique allowed the resolution of single protein isoforms, or proteoforms, into single ‘spots’ in a polyacrylamide gel, allowing the quantitation of changes in a proteoform′s abundance to ascertain changes in an organism′s phenotype when conditions change. In pursuit of the comprehensive profiling of the proteome, significant advances in technology have made the identification and quantitation of intact proteoforms from complex mixtures of proteins more routine, allowing analysis of the proteome from the ‘Top-Down’. However, the number of proteoforms detected by Top-Down methodologies such as 2D-PAGE or mass spectrometry has not significantly increased since O’Farrell’s paper when compared to Bottom-Up, peptide-centric techniques. This article explores and explains the numerous methodologies and technologies available to analyse the proteome from the Top-Down with a strong emphasis on the necessity to analyse intact proteoforms as a better indicator of changes in biology and phenotype. We arrive at the conclusion that the complete and comprehensive profiling of an organism′s proteome is still, at present, beyond our reach but the continuing evolution of protein fractionation techniques and mass spectrometry brings comprehensive Top-Down proteome profiling closer.

## 1. Introduction

The mere mention of the phrase ‘Top-Down Proteomics’ is likely to incite some strong and varied opinions from proteomics researchers. This is mainly due to the lack of a precise definition of what the ‘Top-Down’ analysis of a protein actually means. The term Top-Down came about to distinguish protein identification using intact protein isoforms, or proteoforms [1], from the very widely used term ‘Bottom-Up’ [2], that is used to describe the analysis of a proteome by first enzymatically digesting all of the proteins into peptides and subjecting them to a ‘shotgun’ analysis (a term first used in DNA sequencing [3,4]). Top-Down was coined by those seeking to identify intact proteins using a mass spectrometer [2,5], performing no enzymatic digestion events prior to introducing the proteoform molecules into the mass spectrometer. However, this use of the term ‘Top-Down’ ignores a large number of techniques that also allow the analysis of a proteome from intact proteoforms.

The definition of Top-Down proteomics that we will adopt here places more weight on the quantitation of intact proteoforms and less on the need to identify the proteoform as an intact molecule, allowing the isolated proteoforms identification through its peptides. Shotgun methods require that a peptide be identified and assigned to a gene product, the amino acid sequence that is bioinformatically determined from a gene [6], before quantitation is performed using a limited number of peptides to infer the abundance of a gene product rather than a proteoform. Here, we define Top-Down as the quantitation of an intact proteoform before its identification. At first glance this may seem odd but it revolves around performing proteome analysis by fractionating intact proteoforms until single proteoforms are isolated, a workflow routinely performed in 2D-PAGE fractionation but also able to be achieved through multiple, orthogonal methods of chromatography. Quantitation using 2D-PAGE is then able to be carried out prior to identification through the densitometric analysis of the gel. Differences in the intensity of the same spot in replicate gels of the samples being compared is used to determine which spots are changing in abundance without the need for identification. Once the spots that are altered in abundance are determined, the protein within that spot can be digested to peptides to release them from the gel thereby allowing for mass-spectrometric analysis and subsequent identification of only the proteoforms altered in abundance rather than all proteoforms. However, using this technique and a robotic spot cutter, a researcher could analyse every visible spot in a gel and therefore the majority of detectible proteoforms, at the cost of an extremely large amount of instrument time, although this concept will be explored in a later section in more detail.

LC-MS/MS analysis of intact proteins can also resolve to proteoforms, where the same deconvoluted mass, derived from the mass-to-charge ratios (*m*/*z*) of different molecular charge states of the proteoform, is detected at an identical chromatographic retention time in different samples. In reality, even after three dimensions of orthogonal separation [7] (charge by liquid IEF, molecular mass by continuous elution SDS-PAGE, and hydrophobicity by reversed phase chromatography), proteoforms can still co-elute and be simultaneously ionised. The ability of the MS to determine the deconvoluted proteoform mass from the isotope series of a single charge state and select only the molecules of the ion for fragmentation while excluding all other ions entering the MS makes the ionisation of multiple proteoforms at the same time less of an issue. The key point of contention is whether Top-Down proteomics requires ions of intact proteoforms to enter the MS for analysis or whether the purification of a proteoform to homogeneity, prior to the proteoforms enzymatic digestion and identification by MS analysis of the resulting peptides is also Top-Down proteomics. It is our firm belief that both approaches can be considered Top-Down proteomics and this review aims to define the current employed methodologies for the system-wide analysis and quantitation of intact proteoforms rather than analysis and quantitation through inferring the presence of a protein through its peptides.

## 2. Why Analyse Intact Proteoforms?

Despite the fact that mass spectrometers continue to rapidly increase in speed, sensitivity and dynamic range, the most common implementation of the technology, the detection and quantitation of peptides to infer changes in the abundance of gene products, has to be acknowledged as flawed. Many reports prioritise throughput of biological and technical replicates at the expense of both deep proteome coverage and high sequence coverage of individual proteoforms. While the sampling of a proteome in a single analytical run, such as the work of the Mann [8,9,10] and Coon [11] groups is potentially very useful for providing rapid and comprehensive data on the abundance of gene products, a significant proportion of the proteins ‘identified’ are actually inferred by a small number of peptides. In this case, we refer to gene products as the translated protein sequence that is in the database being searched, which may not contain entries for transcript or splice variants as those are not encoded at the genome which is being sequenced. While the reporting of protein identifications by a single peptide is generally discouraged by proteomics journals [12], it appears to be entirely arbitrary that the detection of two peptides is considered a definitive identification, especially if those peptides are not proteotypic. This is particularly relevant for the identification of the products of small open reading frames or smORFs, which are difficult to identify by bioinformatics and mass spectrometry and have been shown to have a high rate of essentiality in bacteria [13]. In shotgun-based LC/MS/MS proteome analysis, protein inference is a term that arose in relation to an inherent problem with this technique and other methods that create peptides from a mixture of proteoforms. The problem expands further when the peptides are then further fractionated, completely disconnecting them from their parent proteoform. Protein inference is concerned with deconvoluting where peptides originated from [14,15]. In other words, which proteoform does a particular molecule of a peptide belong to? This very clearly makes the point that shotgun methodologies are identifying the presence of gene products rather than proteoforms.

Reviews on the subject of comprehensive proteome coverage by shotgun techniques refer to literature that provides direct evidence for the protein translation of 90% of human genes, referring to ‘gene products’ [16], rather than proteoforms. While this gives the impression that proteomics as a field is nearing the ability to definitively analyse human biology at the molecular level, it overlooks the necessity for proteomics to identify and quantify proteoforms which are often significantly different to the gene from which it was first translated. The starkest case in point is the twin publications of drafts of the human proteome in Nature in 2014 [17,18]. Wilhelm et al. [17] report the combining of ~17,000 LC-MS/MS experiments into a dataset that provides peptide evidence for 92% of genes listed in SwissProt (18,907/19,629) but only 22% of the proteoforms listed (19376/86771). No discussion is made about how many peptides identify each gene product. Kim et al. [18] supply this information (extended data Figure 1b) with less than 5500 gene products, or ~32% of the total detected gene products, described by less than 5 peptides with the overall median sequence coverage being ~28% which, in our opinion, cannot be considered a comprehensive analysis of a proteome. Other researchers have a similar opinion such as Ezkurdia et al. [19] who reanalysed the data of Kim et al. after noting the presence of peptide evidence for genes of olfactory origin, when nasal tissue was not sampled. Ezkurdia et al. point out that neither study distinguishes between discriminatory, or proteotypic, and non-discriminatory peptides.

Astute readers will therefore understand this review′s emphasis on performing identification and quantitation on intact proteoforms rather than gene products. The currently employed strategies and methodologies for Top-Down proteome analysis identify 1000–1500 proteoforms [20,21]. This is more than 10-fold lower than the human proteome drafts and is due to the combination of the dynamic range of proteoform concentration within the proteome and the analysis techniques of 2D PAGE and mass spectrometry being concentration sensitive techniques that require significant fractionation of the proteome in order to detect low abundance proteins [22,23]. The fractionation required for comprehensive proteome analysis creates sample numbers that are beyond the resources and willingness of most labs to analyse. The result of this is that it is rare that every detectable proteoform in a sample is actually identified. Spots from 2D-PAGE are often ignored or not able to be reliably quantitated, while ionised proteoforms may not produce sufficient sequence ions for reliable identification.

The work to optimise 2D-PAGE methodologies by the Coorssen laboratory has shown that ~3000 protein spots can be resolved from 100 µg of total mouse brain protein by separating soluble protein and membrane protein fractions, using infrared detection of coomassie blue stained proteins and a deep imaging strategy that excises high abundance proteins prior to reimaging of the gels [21]. This work employed the smallest commercial IPGs available, 7 cm pH 3–10 non-linear gradients. The mouse genome is estimated to have 20,210 coding genes (UniProt proteome ID UP000000589) meaning ~15% of gene products could be represented in this data but it is likely far less when many ‘spot trains’ are in reality related proteoforms that differ by post-translational modifications (PTMs) that alter their isoelectric point (p*I*), such as phosphorylation. Further evidence for this being correct is shown in the work of Pieper et al. [24] who analysed the human serum proteome using 2D-PAGE after sample fractionation using immunodepletion to remove the eight most abundant proteins (and any proteins bound to those) prior to anion exchange chromatography and subsequent size exclusion chromatography of those fractions. The 66 fractions produced were then each subjected to 2D-PAGE, resolving ~3700 spots of which 1800 could be identified by mass spectrometry. These 1800 identifications could be collapsed into 325 proteins or gene products, representing ~1.6% of the possible human products.

Top-Down MS similarly reports the identification of ~1000–1500 proteoforms (In this case, a distinct proteoform is defined by a high accuracy measurement of the masses of the charge states and isotope series of the proteoform′s ions to calculate the deconvoluted mass. Identification of the proteoform is through the observation of sufficient sequence ions from fragmentation of one the molecules of one (not all) of the charge states to identify the gene product. The proteoform′s measured mass may vary from the calculated mass of the gene product and thus the nature of the modification can be inferred from the mass difference. Thus, Top-Down MS should give an accurate number of proteoforms as the sequence ions identify the gene product and the intact mass measured defines the proteoform. However, the number of charge states increases with proteoform size as the number of amines and protein length increases [25] and the number of isotopes and thus observed peaks (if the mass spectrometer′s resolution is high enough) increases within a charge state as more C-13 atoms are present in larger proteins [26]. As the number of molecules of a proteoform being ionised are spread over numerous charge states and only a single charge state ion can be selected for fragmentation in current instruments, sensitivity is reduced when compared to performing mass spectrometry on peptides that are most often in a single charge state. The solution to this would be to ‘force’ all of the molecules of a proteoform into a single charge state within the instrument′s mass range, measuring the mass by deconvolution of the isotope series of that charge state, and then performing fragmentation on the ions of the single charge state. One solution to this challenge is termed “super-charging” which involves a solvent additive prior to electrospray ionisation to increase ion charge states. The first of these additive compounds were *m*-nitrobenzyl alcohol or sulfolane [27,28], which sought to increase the number of charges of every ionisable molecule to the theoretical maximum, to increase signal and sensitivity. More recent development of this technology has gone beyond the theoretical maximum charge for model proteins, using carbonate additives such as 1,2-butylene carbonate [29]. This additive compound allowed a dramatic increase in sequence coverage of model proteins to 85%–95% up to a size of 66.5 kDa. These additives are yet to be used with chromatographic fractionation but if compatible could great improve proteome coverage using Top-Down MS because of the increase in sensitivity gained by having more protein ions in a small number of charge states. However, the smaller number of charge states will require mass spectrometers of even higher resolving power which are currently beyond the resources of most facilities, a fact to be further addressed in a later section.

In 2016, the pursuit of quantifying proteome changes has meant that a comprehensive analysis is equally reliant on electrophoresis, both in polyacrylamide gels and in liquid, chromatography and mass spectrometry. With the current technical limitations and the concentration sensitive nature of the applied techniques, the comprehensive profiling and quantitation of proteome changes is still reliant on the application of unbiased fractionation techniques that reduce sample complexity and increase the concentration of low abundance proteoforms to a level that allows analysis with high sequence coverage and thus reliable quantitation. However, it is necessary to remember that increasing the number of fractionation techniques can lead to unintended loss of sample and requires a considerable amount of sample that may not be obtainable.

## 3. Defining ‘What’ Proteome Analysis Actually Is

With the current limitations imposed by technology and methodology, and researcher′s understandable unwillingness to devote weeks or months of analysis time to a single sample, there remains a question as to the future direction of whole proteome analysis. In the experience of our Core Facility, we can define this as the ‘what do you want out of life?’ question. The first option is the complete characterisation of the entire proteome of a cell, or the detection of every single proteoform and its characteristic PTM, something that is beyond our current reach. Our extensive work on Mycoplasmas, an organism with a genome of ~893 kb with less than 700 predicted ORFs, has shown that the most highly expressed ORFs are extensively proteolytically processed into multiple proteoforms meaning that this so-called ‘simple’ organism produces many thousands of proteoforms from its reduced genome. The inability to fully characterise this proteome further demonstrates that complete proteome characterisation is currently unachievable [30,31,32].

Alternatively, proteome analysis could also be defined as simply determining the difference in the abundance of particular proteoforms through performing differential display, where only the detectable proteoforms that are altering in abundance need to be identified. Thus, changes in biology are defined by changes in the abundance of a particular proteoform rather than the abundance of a gene product. This could necessitate unbiased sample fractionation to increase the depth of analysis and requires careful experimental design to minimise sample losses.

**Figure 1:**
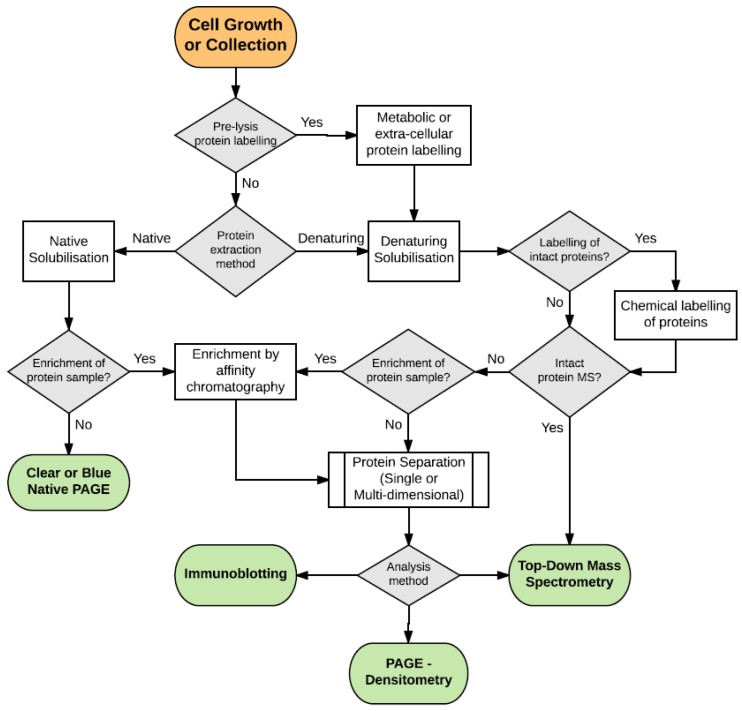
Common workflows for Top-Down analysis. The grey box represents the start point of experimental design, the collection of clinical, environmental samples or tissue/cell culture including growth conditions for treated samples with controls. At this point, proteins can be labelled during tissue or cell culture for later quantitation if desired, for example by 15N amino acids or Stable Isotope Labelling of Amino acids in Cell culture (SILAC). For enrichment/extraction of surface proteins, protein biotinylation can be performed on washed cells before cell lysis and protein extraction. Native protein extraction is performed to maintain their physiological associations and/or in their structural conformations. Native protein lysates may be analysed by Blue or Clear Native PAGE to gain insight about protein complexes and interactions. Alternatively, denaturing buffers and surfactants can be used to solubilise as many proteins as possible without retaining their secondary and tertiary structure. The sample may then be enriched for proteins of interest using techniques such as affinity chromatography to extract a subset of proteins from the sample (e.g., only those proteins capable of interacting with the host molecule heparin). Once extracted, the soluble proteins may also be chemically labelled for relative quantitation of samples by fluorescence (e.g., Differential In Gel Electrophoresis (DiGE)) or mass spectrometry (e.g., isobaric Tagging for Relative and Absolute Quantification (iTRAQ) or Tandem Mass Tags (TMT)). The proteins are often separated to homogeneity by Isoelectric Focussing and/or PAGE, which may be followed by densitometry, mass spectrometry or immuno/western blotting analysis. Boxes in green represent possible endpoints of sample analysis.

For the purpose of this review we will define proteome analysis as any method which seeks to identify, with or without quantitation, the range of detectable proteins and proteoforms from a biological sample in a defined point of time. The selection of technique and workflow very often comes down to limitations of the sample, time and financial costs as well the experimental aim or hypothesis to be tested. Common workflow choices are outlined in Figure 1 and the range of techniques that could be utilised in this workflow is outlined in Table 1.

## 4. Two-Dimensional Gel Electrophoresis Using Isoelectric Focusing in Immobilised pH Gradients and SDS-PAGE

As stated elsewhere in this issue, what is referred to as 2D-PAGE was first reported by Patrick O′Farrell in 1975 [39]. The use of tube gels and ampholytes to establish the pH gradient in the first dimension of isoelectric focusing (IEF) required considerable technical skill and suffers from pH gradient instability and drift, making it difficult to create reproducible gel images [40,41]. This changed dramatically in 1982 with the introduction of immobilised pH gradients (IPG) by groups led by Angelika Gorg and Pier Georgio Righetti [42,43]. The stability of the pH gradient along with the reproducibility and convenience provided by commercial production of IPG strips made 2D-PAGE the most highly resolving technique for fractionating proteomes available and one could argue that this is still the case in 2017. However, there is still a regular flow of articles that reinforce an old “myth” by proclaiming that 2D-PAGE is challenging, has poor reproducibility, has difficulty with hydrophobic proteins, membrane proteins and proteins at the extremes of the pH range. It is likely that these ‘myths’ came about from people handling large format 18 or 24 cm gels, but these ‘myths’ have been proven to be incorrect through a series of articles adequately establishing the techniques′ reproducibility [44,45,46,47,48]. The vast majority of problems encountered in 2D-PAGE are historically the result of poor sample preparation, or more simply having a sample that contains molecules that are not proteins. A great deal of work has been performed that demonstrates that the correct use of chaotropes, surfactants, ampholytes, the complete reduction and alkylation of cysteine bonds and the removal of all salts and conductive non-protein species is necessary for the creation of well resolved gels [49,50]. The amount of protein loaded and the dynamic range of concentration must also be carefully controlled to ensure reproducibility. It must also be acknowledged here that it is possible that two proteoforms differing by an amino acid substitution that does not alter protein charge, such as a glycine to alanine substitution, may not be able to be resolved into two distinct spots. To our knowledge this has not been empirically tested. However, a biochemistry textbook also contains the knowledge that the pKa of an amino acid′s side chain is determined by the amino acids around it, so in the above scenario, the resolution of both proteoform′s might be possible. If the researcher is fortunate enough that the peptide defining each proteoform is detected by the MS after in-gel digestion, a false negative result will be avoided. One could argue that Top-Down MS analysis is the solution to this issue because, even if the proteoforms are ionised into the MS at the same time, the resolving power of an FTICR-based MS would reveal two distinct masses for the two proteoforms. However, there are a number of combinations of two or three amino acids that have almost identical masses, such as DT and ES both summing to 216.0746 Da and NT, QS, AGS and GGT all summing to 215.0906 Da [51]. These proteoforms would not be able to be resolved by current MS instrumentation. Ultimately, this reinforces the notion that proteome analysis should not be carried out by a single technique.

While the most common extraction methodology observed in the literature is still to disrupt the sample in the presence of 8 M of the chaotrope urea and 4% of the zwitterionic surfactant CHAPS, this will undersample the proteome in question. Sample disruption is a critical step, especially for bacterial [48] and plant samples [52] with rigid cell walls, however the frozen disruption method used for these samples is equally applicable to tissue samples and results in higher proteome yields [53]. The production of a fine ‘talcum-like’ powder then allows a far higher extraction of proteins most likely due to the increased surface area and thus accessibility of the proteins. Protein extraction can then be performed using solutions of increasing solubilisation power [54], such as low molarity Tris-HCl or PBS followed by a surfactant and chaotrope mixture (urea, thiourea and zwitterionic surfactant) and finally boiling the remaining insoluble material in SDS, a simple route to reducing proteome complexity. However, protein extraction can be performed in more ways than there is space in this manuscript to include, with the aim of either solubilising as many proteins as possible in one step or sub proteomes using different surfactants [44,55,56,57,58] or solvents [46,59]. It is at this point that reduction and alkylation of cysteine should be performed to ensure the presence of single proteoforms. Phosphine-based reducing agents are the best choice as they only react with disulphide bonds and not with alkylating reagents as thiol-based reducing agents [60].

The aim of isoelectric focusing of proteoforms is to resolve all of the molecules of each proteoform in the narrowest space in the IPG strip at the proteoform′s isoelectric point, or the pH at which the molecules of a proteoform have a net charge of zero. This means that any solubilising reagents that can alter the protein′s charge, such as SDS, cannot be present. In addition, the low conductivity of proteins means that effective performance of IEF relies the IPG being subjected to extremely high voltages or field strengths (up to 10,000 volts) at extremely low currents (as low as 1 μA) [50]. Thus, denaturing proteome extraction has relied on the chaotrope urea since the 1970′s [61,62] at a concentration between 7–9 M, later being supplemented with 2 M thiourea [63,64], and surfactants to disrupt the association of lipids and help maintain protein solubility during IEF [50,65]. The mechanism by which urea disrupts protein structure is controversial, with theories that it either disrupts the water structure around and within the protein, weakening hydrophobic interactions and making hydrophobic residues less compact and more readily solvated, or that urea interacts with the protein directly through stronger electrostatic interactions or preferential van der Waals attractions [66].

Surfactants, or detergents, are more straight-forward in their mechanism of protein disruption. Surfactants typically consist of two distinct regions in their molecular structure, a hydrophobic region of long hydrocarbon chains with no ionisable groups that interacts with the protein and an ionisable group that interacts with solvent molecules such as water. For use in IEF, surfactants must be zwitterionic or contain both a positively charged group, typically an amine, and a negative charged group, typically a sulfoxide, that can interact with surrounding solvent but the charges cancel each other out and the net charge on the molecule is zero. This means that when bound to a protein molecule, the zwitterionic surfactant does not alter the molecules isoelectric point but enhances solubility by interaction with solvent molecules that the protein cannot perform as changes in pH during focusing cause amino acid side chains to become charged or neutral, altering their solvent interactions. CHAPS is the most commonly used surfactant more than 30 years after its introduction [67,68], but numerous other amidosulfobetaine-based surfactants are available that can be considered to have greater solubilising power [56,58,69]. Most core facilities will have their preferred protein extraction protocol as an initial attempt with a new sample but it is common in our facility and others to alter the extraction conditions [70], sample permitting, to improve extraction or alter the proteins being extracted.

One overlooked aspect of sample preparation is the need to reduce disulphide bonds and prevent their reforming by alkylating the resulting thiol. In 2D-PAGE, this is necessary to ensure that individual proteoforms resolve to the correct pI while in LC-MS/MS it is important to ensure that disulphide bonded dipeptides are not being selected and fragmented. As a result, the measured parent mass of the dipeptide will be significantly larger than either peptide and the MS/MS spectrum will contain fragments from both peptides, the result being that the spectrum will remain unmatched upon database searching. By far the most popular reagent for reduction of disulphides is dithiothreitol (DTT) at a relatively high concentration of ~20 mM. This is necessary because DTT is itself a thiol and will react with the reagents subsequently used to alkylate the protein thiols. It is for this reason that reduction and alkylation of proteins with DTT is a two-step process with DTT treatment for ~30 min followed by alkylation for a further ~30 min, most commonly by iodoacetamide at double the concentration of DTT to ensure the protein thiols are alkylated. However, a simple alternative exists using phosphine-based reducing agents which only react with disulphide bonds and not reagents used for alkylation. This allows reduction and alkylation to be performed in single step and has also been shown to improve spot resolution [60]. The need to alkylate and block reduced protein thiols comes from the observation that spot resolution is improved [71,72], cysteine containing peptides are detected more frequently [73] and that cysteine can undergo beta-elimination at alkaline pH which can subsequently cleave peptide bonds [71]. This artefact is completely eliminated by alkylation. Iodoacetamide is still the most commonly used alkylating reagent, but is light sensitive [74] and the reaction must be performed in the dark. A simple and cheaper alternative is to use acrylamide monomers, which are not light sensitive and had been observed as alkylation ‘artefacts’ in SDS-PAGE [75,76]. While discussing artefacts, some researchers cite carbamylation of lysine as an issue when performing protein extraction and solubilisation in solutions of urea. The work of McCarthy et al. [77] showed that carbamylation only occurs if the sample is left at high temperatures (>50 °C) or for long periods of time (>48 h) and during IEF, the cyanate ions are removed by the electric field and modification does not occur.

One key limitation of 2D PAGE is that if a whole sample is analysed on a single 2D gel then the number of spots, or proteoforms, that are observable on that single gel is likely to be less than 15% of the gene products predicted by the genome. If the researcher is only concerned with proteoform abundance changes in the highest abundance proteoforms, then a single 2D gel is likely to be acceptable. However, if one is trying to assess not only the total number of gene products in a proteome but the range of proteoforms, a single gel is inadequate as simply loading more sample will reach the dynamic range of concentration, a point well-illustrated by a figure by Anderson showing the dynamic range of proteins in human serum [78]. The solution to this is the unbiased pre-fractionation of the sample prior to IEF on the IPG which is reliant on having sufficient sample available.

A great number of technical advances were made in the areas of sample preparation and pre-fractionation [79,80] to address the problems of the dynamic range of protein concentration. However, despite the central nature of this issue to all proteome analysis techniques there is a growing trend that it is ignored in the quest for high throughput as mass spectrometer manufacturers push to increase the dynamic range of their instruments which will need to span 10 orders of magnitude rather than the current 4–6 orders [81]. Pre-fractionation techniques are universally applicable to a protein sample from any source (plant, bacteria, tissue etc.) and can be performed prior to any downstream analysis technique, not just 2D-PAGE, as demonstrated by the three dimensions of fractionation (liquid IEF, preparative SDS-PAGE and RP-HPLC) employed by the Kelleher group for Top-Down MS analysis [7]. In practice, the fractionation of a sample causes the exponential increase in the number of 2D gels or LC-MS/MS runs that need to be performed to analyse the generated fractions. In the case of 2D-PAGE being performed for differential display through the use of smaller format 7 or 11 cm gels, the increase in costs is minor whereas using LC-MS/MS the increase in cost due to instrument time is significant as every fraction needs analysis before quantification can be performed.

Sequential extraction by solubility is the simplest fractionation technique [54] where, following disruption, the sample is suspended in a physiological buffer, such as PBS or Tris-HCl, and then centrifuged or further ultracentrifuged to obtain membranes [82]. The pellet is then resuspended in a solution containing chaotropes and surfactants before being centrifuged again. Any remaining pellet is then boiled in an SDS-containing buffer to solubilise the most recalcitrant proteins. Using this approach on whole paralysis ticks resulted in a doubling of observable spots in 2D PAGE of the separate Tris and chaotrope solubilised fractions with quite different spot patterns [83].

An alternative prefractionation technique prior to IEF in IPGs is the use of liquid phase IEF using either a Microrotofor (Bio-Rad; [84,85]), ZoomIEFRunner (Thermo; [86,87]) or OFF-GEL (Agilent, [88,89]). In all of these devices, proteins are focused by p*I* into separate ‘chambers’ where they can be removed and analysed separately. Successful fractionation in either of these devices requires the same conditions as focusing in IPGs in that the sample needs to be as clean as possible in a solution of 7 M urea, 2 M thiourea, minimal surfactant (such as ≤ 1% C7BzO) and a minimal amount of carrier ampholytes to assist solubility. A note of caution when performing SDS-PAGE with samples containing ampholytes. Although very small molecules, it has been observed in our laboratory and others that ampholytes of basic pH migrate very slowly through SDS-PAGE and bind common protein stains, obscuring any protein bands or spots up to a ‘mass’ of 20 kDa in samples containing ampholytes with a p*I* > 9 (unpublished observation).

The Rotofor uses carrier ampholytes to create the pH gradient which is divided into 12 ‘chambers’ by a permeable membrane, thus creating 12 fractions. While providing highly resolving separation for ~1 mg of protein, the need for adhesive “scotch” tape to seal the rotofor chamber means that the device can be difficult to set up without leaking thereby affecting reproducibility and reliability. The ZoomIEFRunner uses acrylamide membranes of defined pH to separate chambers (up to 7), providing a very robust separation platform that is more expensive per sample but more reliable. The OFF-GEL uses an IPG strip to which is mounted a series of chambers spanning the length of the IPG, much like using multiple sample cups for IPG loading. These chamber cups are filled with a chaotropic solution and fractionation is reliant on protein molecules moving from chamber to IPG by the electric field, focusing to their p*I* in the IPG and then diffusing from the IPG back into the solution in the chamber above the p*I*. While effective, we have noticed that there is a considerable amount of protein left in the IPG after solutions are harvested from the chambers. This can be remedied by dissection of the IPG and adding the relevant section to the recovered solution to passively diffuse any remaining protein from the gel prior to SDS PAGE.

The true power of this fractionation is observed when the proteins in the p*I* fractions are resolved on an IPG spanning only the pH range of the p*I* fraction. For example, focusing a sample on a 7 cm IPG strip with a range of pH 3–6 increases the resolving power by 2.3-fold (Bio-Rad). However, if an unfractionated sample was run on this range IPG strip, there would be a wall of protein stacked at the pH 6 end of the strip containing all of the proteoforms with a p*I* of 6 and above. The sensitivity is not increased in this case as much of the loading capacity of the IPG strip is taken up with proteoforms that don′t focus within the IPG strip′s range and the focusing and resolution of spots is compromised by these molecules at the end of the strip. Thus, it is better to fractionate the sample with the above-mentioned devices and utilise the loading capacity of the IPG strip by only applying proteoforms that focus within that range which will boost sensitivity and reveal more proteoform spots and increase the number that can be properly quantitated.

A prime example of the value of 2D PAGE is highlighted in the *M. hyopneumoniae* surface adhesin protein P135. The gene encoding P135 is predicted to produce a protein of approximately 135 kDa however, this protein is rarely identified by LC-MS/MS analysis of tryptic peptides derived from proteins that resolve at this molecular mass by 1D PAGE [90]. Instead, peptides matching to the entirety of the P135 gene product, termed the “pre-protein”, are identified in protein bands that resolve at approximately 50 kDa [90]. Although these proteins have a similar molecular mass, they possess distinct isoelectric points, whereby the individual fragments were only resolved when separated by 2D PAGE. Trypsin digestion and subsequent LC-MS/MS analysis of these spots verified that these spots were distinct proteoforms of the P135 pre-protein, produced as a result of endoproteolysis. These endoproteolytic cleavage events occur at TTKF↓QE motifs that were identified using a combination of Edman degradation sequencing and the identification of semi-tryptic peptides [90]. Edman degradation sequencing involves the adsorption of a peptide to a surface, followed by the labeling of the *N*-terminus of the peptide with phenylisothiocyanate [91,92]. Following this, an anhydrous acid is added to selectively detach the labeled *N*-terminal peptide which can then be identified using chromatography. This process is then repeated until the desired protein sequence has been identified. This technique has long been the gold standard of protein sequencing however it is not without its limitations. One of the major limitations being that if the peptide sequence contains an *N*-terminal modification/PTM, binding of phenylisothiocyanate is blocked; rendering sequencing impossible. This was demonstrated in the analysis of the endoproteolytic cleavage site in P135 that generates the central cleavage fragment, P48. The *N*-terminus of this fragment contains a pyroglutamate in place of a glutamate, explaining why Edman degradation sequencing failed in this instance. The true *N*-terminus of this fragment was only identified through the identification of a semi-tryptic peptide corresponding to the true *N*-terminus [90]. A semi-tryptic fragment is defined as “peptides which are cleaved at the *C*-Terminal side of arginine (R) and lysine (K) by trypsin at one end but not the other” (Proteome Software [93]). Thus, in the case of identifying endoproteolytic sites, semi-tryptic peptides that do not begin after an arginine or lysine residue can be inferred to be the site of cleavage that occurred during protein maturation in the organism.

Post-translational modifications generated by endoproteolysis, pose a unique challenge for the identification of defined cleavage fragments and proteoforms. Edman degradation of a purified proteoform provides direct evidence of endoproteolysis, but the process has low throughput and Edman sequencers are a rarity (but highly prized and eagerly maintained by a small number of specialised researchers). However, for the identification of endoproteolytic cleavage events that occur natively within a cell, Bottom-Up proteomic techniques on their own are not suitable due to the requirement of digestion of proteins to peptides. Although labelling techniques exist to identify endoproteolysis by Bottom-Up mass spectrometry, such as COFRADIC [94] and more recently popularised by reductive dimethylation with peptide enrichment [95], the MS/MS data is not always as convincing as it needs to be. Top-Down methodologies can remove the ambiguity of these data by achieving proteoform resolution of cleavage products as demonstrated in the P135 example outlined above.

## 5. Blue and Clear Native PAGE

Whilst 2D PAGE offers a great deal in terms of fractionating individual proteoforms, certain limitations remain within these conventional methodologies. In particular, biological context of protein-protein interactions and complexes are lost during denaturing sample preparation and in both separation dimensions. Furthermore, some hydrophobic membrane proteins can be lost through precipitation during the first dimension of standard isoelectric focusing, although a great deal of work has been done in this area [44,96].

The interactions and complexes formed between proteins are responsible for most molecular processes and vital cellular functions, such as DNA replication, transcription and mRNA translation, cell signalling, and metabolic, transduction and differentiation pathways [97]. These biological processes are precisely coordinated and regulated by dynamic signalling networks of interacting proteins. Accordingly, their analysis is essential to expand our knowledge. Unlike conventional denaturing methods, the purification of interacting proteins must be performed using conditions that preserve their native environment to maintain the relevant protein interactions. The Schägger lab [36] developed native electrophoresis as a single step isolation technique in the preparation of mitochondrial membrane protein complexes. This procedure has since been utilised to derive intact protein complexes from various membranes [98], tissue or cell lysates [36] from eukaryotes and prokaryotes [99]. This preserves the protein′s oligomeric states allowing for the native protein masses to be determined (up to 10 MDa) as well as retaining native functions. There are numerous methods available for the detection and purification of stable protein-protein complexes which associate through strong interactions. Conversely the detection of very weak or transient protein-protein interactions remains a difficult task. Transient interactions are expected to control the majority of cellular processes [100], but they are temporary in nature and typically require a specific set of conditions that promote the interaction to fulfil their biological function in vivo. Maintaining these interactions subsequent to extraction demands stringently controlled variables and handling up to and including fractionation.

Blue native and clear native polyacrylamide gel electrophoresis (BN or CN-PAGE) allows for the study of intact and complete protein complexes or transient protein-protein interactions [36]. Sample preparation for the isolation of intact protein-protein complexes into a soluble phase requires the use of mild non-ionic or zwitterionic detergent conditions dependant on the types of interactions to be preserved [101]. Digitonin [102], Triton-X 100 [103], C7Bz0 and dodecylmaltoside [36] are widely used to maintain various levels of transient, hydrostatic, electrostatic and stable protein-protein interactions, in cold Tris-based or PBS buffers of physiological pH to conserve heat liable complexes. The use of detergents here is not to denature or act as charge modifiers as in the case of denaturing SDS conditions. Rather the introduction of Coomassie Blue G-250 or Deoxycholate, respectively giving the Blue or Clear nomenclature, into the sample and cathode buffer sufficiently coats the proteins and imparting an overall negative charge to the complex allowing its separation according to relative size in the gel. The inclusion of Coomassie Blue G-250 is ideal for a high resolution separation of stable protein-protein interactions such as membrane complexes, however it can act as a detergent in some circumstances and disrupt a proportion of transient interactions [102]. Thus, clear native preparative steps are best applied to capturing weaker interactions in hydrophilic and acidic protein complexes.

A number of additions or variations to the Blue or Clear native procedures have been developed to further resolve the retinue of interacting protein complexes via a Top-Down workflow. The addition of secondary and tertiary dimensions can be used to reduce sample complexity and increase resolution. Subsequent to the first native electrophoretic separation, a second dimension can be employed for further fractionation by dissociating the components of the complex. Individual lanes from the Blue or Clear native gels can be excised, soaked in 1% SDS and 2-mercaptoethanol, allowing the denaturation of complexes within the excised gel strip, which is then rotated 90° and laid in the horizontal plane onto a conventional SDS PAGE akin to the IEF strip in 2D PAGE. The second-dimension electrophoresis separates the complexes into its components by mass, allowing for the isolation and mass spectrometric determination of intact proteoforms which can be subsequently identified by in-gel digestion and mass spectrometry. An example of this work flow was the characterisation of the glutamyl aminopeptidase MHJ_0125 from *M. hyopneumoniae* [104]. After solubilisation in the presence of dodecylmaltoside, analysis was performed by 2D Clear Native PAGE using sodium deoxycholate as the charge modifier, revealing that the 40 kDa monomer of the protein exists as a 12 unit homo-complex in vivo in the first dimension, similar to other glutamyl aminopeptidases, before appearing as a single spot at 40 kDa after complex denaturation and SDS-PAGE in the second dimension. In addition, the complex was shown by CN-PAGE to spontaneously assemble when the monomer is expressed in *E. coli*.

2D Blue Native procedures (with two native dimensions) can also be used to isolate intact supercomplexes in digitonin in the first BN dimension. The excised gel lane can be subsequently treated with DDM which will disrupt supercomplexes whilst maintaining their subsidiary stable complexes [105]. This alteration in detergent type between the two dimensions exceptionally separates complexes of similar electrophoretic mobility without losing native structures. The secondary dimension here can be excised and coupled with the denaturing gel producing a 3 dimensional separation of proteoforms comprising the complex.

Continuing with the so-called 3D separation techniques, native IEF using a rotofor in a solution of ampholytes and zwitterionic mild surfactants to fractionate complexes by isoelectric point has been performed [84], the fractions of which can then be applied to conventional BN or CN PAGE, or SDS-PAGE. Laser-induced liquid bead ion desorption-MS of protein complexes from blue-native gels was developed to eliminate the second dimension PAGE and to directly produce a reproducible Top-Down system identifying large proteins and complexes whilst being tolerant of detergents [106]. Although this method would be ideal in fast tracking a robust Top-Down isolation of proteoforms in protein complexes, it is currently prohibitively expensive. The assembly of protein complexes and membrane protein interactions can also be analysed by mass spectrometry using modified instrumentation [107,108,109], but as this requires prior knowledge of the protein or complex and its purification to homogeneity, it is beyond the scope of this review.

## 6. Label-Free and DiGE-Based Relative Quantitation in PAGE

Relative protein quantitation by Western blotting is a well-established and highly specific method of obtaining crude, relative quantitative data on a single proteoform between samples [110]. As it is not a proteome-wide technique, its usefulness for analysing the variety of changes in protein abundances in most biological responses is limited. Furthermore, it requires prior knowledge of the protein of interest to experimentation and relies on the availability and quality of a protein antibody.

Relative quantitation of unique proteoform abundances can also be achieved using label-free densitometry techniques employing computational software. These methods require accurate measurement of total protein by BCA [111], Modified Lowry [112] or Bradford [113] assay and subsequent equal protein loading for two dimensional electrophoresis, usually in 3 or more replicates for each sample to be compared to account for technical variation. The gels are then stained with a fluorescent or colorimetric stain such as SYPRO Ruby or Coomassie Blue and scanned with a high sensitivity and resolution scanner such as GE′s Typhoon FLA 3500. Replicate gels may be manually compared for proteoform presence/absence or with image analysis software to assign relative quantitative values to stained spots containing resolved proteoforms. In addition to the label-free method, quantitation can be performed on separated proteoforms using protein labelling technologies to distinguish signal between samples.

The Differential In Gel Electrophoresis (DiGE) [114] method utilises covalent derivatisation (labelling) of specific chemical groups present in proteins within comparable samples with up to 3 differential fluorophores (Cy2, Cy3, Cy5) [115]. The dyes react via either a maleimide group with cysteine residues or through succinimide reacting with lysine residues in the protein sample. These dyes have net zero charge and identical molecular weights, so there is minimal alteration to the isoelectric points or size of the labelled proteins. The protein samples to be compared are then mixed together in equal ratios and then separated by two-dimensional electrophoresis on a single gel. The gel is then scanned and the fluorescent signal of the different fluorophores enables detection and relative quantitation of proteoform abundances between different biological samples. This approach substantially reduces the gel to gel technical variability sometimes encountered in label-free densitometry, possibly improving accuracy of protein quantitation between samples. After quantitative analysis, protein spots of interest (differentially abundant) may be extracted from the gel and identified by mass spectrometry. A pooled internal standard can be created by mixing the two samples to be analysed and labelling with the third fluorescent dye. This was found to be a critical step for controlling variability during normalisation of the data, as different normalisation methods are generally comparable. However, care must be taken during biological interpretation of the data as different normalisation methods may change the output of statistically significant proteins [116]. The DiGE method can be used for a variety of samples include tissue sections [117], as well as culture-based methods, and was found to be complimentary to the SILAC quantitation method (discussed later in this article) [118].

## 7. Affinity-Based Separations for the Top-Down Analysis of Complexes and Interactions

Protein-protein interactions underpin almost every aspect of cellular processes that occur within every domain of life, the investigation of which is crucial to understanding these complex systems. There are thus numerous methodologies used for the investigation of protein-protein interactions [119], such as chemical cross-linking [120], two-hybrid screening [121] and affinity purification [122], to name a few. Selecting any of the aforementioned techniques however requires knowledge about the nature of the protein-protein interactions being investigated. For simplicity, protein-protein interactions are often divided into being either stable or transient interactions. Stable interactions are typically permanent interactions that make up multi-subunit protein complexes, while transient interactions are reversible interactions that occur on a temporal basis.

Affinity chromatography offers a simple and relatively cost-effective method for the purification of interacting proteins while also allowing for untargeted analysis to be performed. These experiments are based upon the labelling of “bait” molecule that can be a protein, a mixture of proteins such as the cell surface proteome of a pathogen [32,123], or other molecules such as heparin [31]. This involves the covalent labelling of primary amines such as lysine residues that, due to their positive charge, are often exposed on the surface of native protein structures, making them an ideal target for *N*-hydroxysuccinimide (NHS) ester labelling. These esters are often coupled with an “exploitable” molecule such as biotin [124,125,126,127]. Biotin is a relatively small molecule that shouldn′t disrupt pre-existing protein-protein interactions and the avidin-biotin interaction is the strongest known non-covalent bond which can tolerate a wide range of buffers [128]. Tagging of bait proteins with biotin allows for the immobilisation of these proteins onto avidin allowing vigorous washing and removal of non-specifically associated proteins without removal of the bait proteins.

Affinity chromatography allows for an untargeted method of investigating protein-protein interactions as it does not require any information on potential binding proteins. This method also allows for the identification of proteoforms created via endoproteolysis. The general workflow for these experiments requires maintaining proteins in a native conformation. For this, proteins have been solubilised in 0.5% Triton X-100 in Phosphate Buffered Saline, followed by gentle vortexing and bath sonication [33]. For the elution of bound proteins numerous methodologies have been used, including but not limited to; high concentrations of chaotropes or salts, and low pH [129]. The major limitation of affinity chromatography is the so-called ‘false positives’ attributed to the co-purification of multi-subunit protein complexes. Due to the experiments being performed under native conditions, any proteins that form stable interactions with the ‘interacting’ protein will be co-purified and identified as a being able to bind the bait protein. Targeted binding studies are subsequently needed to confirm the binding interaction.

As mentioned above, affinity chromatography can also be utilised for the enrichment and purification of subproteomes such as membrane fractions and surface proteins. The Djordjevic lab have performed extensive analyses on the surface proteome of *M. hyopneumoniae* using two complementary proteomic approaches: surface biotinylation and trypsin shaving [33,90]. While trypsin shaving is a powerful tool for shotgun approaches, mass context is lost, making it impossible to study proteolytic cleavage. Biotinylation on the other hand, retains the intact proteoforms that can be separated by SDS-PAGE and analysed by mass spectrometry. Due to the sample complexity of solubilised surface proteins, particularly membrane associated proteins, pre-fractionation techniques such as Triton X114 (TX114) extractions can be performed to simplify these samples. In these experiments, *M. hyopneumoniae* cells were biotinylated for 30 s on ice followed by quenching, washing, and protein solubilisation in TX114. TX114 has a cloud point at 37 °C, making it relatively easy to separate proteins that partition to either the aqueous or detergent phase. A TX114 insoluble pellet can also be collected for further analysis; ideally containing integral membrane proteins and other insoluble proteins that can be solubilised in a mixture of chaotropes and surfactants. Following on from this, biotinylated surface proteins can be purified using avidin chromatography. Unlike affinity chromatography used for the purification of interacting proteins, this protocol can be performed under strong denaturing conditions as the biotin-avidin interaction is stable in the presence of detergents and chaotropes. In order to dissociate the biotinylated proteins from avidin a low pH elution is employed that typically contains trifluoroacetic acid in an organic solvent such as acetonitrile [129]. Western blotting using a HRP-conjugated avidin probe is typically used to test the efficiency of the affinity chromatography experiment. This allows for visualisation of the presence of biotinylated proteins in the elutions. This method allows for the enrichment of extremely low abundance proteoforms that would have been overlooked due to the dynamic range of more complex samples.

The P65 lipoprotein is an excellent example of how pre-fractionation, affinity chromatography, and immunoblotting can be used in tandem to visualise subproteomes and to visualise how distinct proteoforms are distributed amongst them. *M. hyopneumoniae* cell surface proteins were labelled with sulfo-NHS-LC-biotin, followed by TX-114 extraction, 1D SDS-PAGE, and immunoblotting with P65 antisera. A distinct banding pattern was detected in each of the aqueous, detergent and whole cell lysate immunoblots with P65 antisera [34]. A dominant band at ~75 kDa can be seen in all lanes; representing P65. Numerous smaller fragments that reacted with P65 antisera could be seen in the aqueous and detergent samples, whereas only the band corresponding to P65 could be seen in whole cell lysates. Lipoproteins that possess transmembrane domains have a tendency to partition to the detergent phase due to their hydrophobicity. This was shown here where a large number of fragments were observed in the detergent fraction. It is speculative to suggest that these fragments represent different forms of P65 that retain an intact lipoprotein attachment site at the *N*-terminus that have been cleaved at the C-terminus. Retention of the lipoprotein anchor located at the *N*-terminus suggests that the TX-114 detergent is needed to extract these proteoform. The fragments in the aqueous phase represent fragments of P65 that were removed from the C-terminus by different cleavage events. Consistent with this hypothesis, the migration patterns of the proteins in both lanes are different. Specifically, this exemplifies the power of combining pre-fractionation techniques for the superior enrichment of low abundance cleavage fragments. Duplicate samples that were separated by 1D PAGE, in-gel trypsin digested and analysed by LC-MS/MS provided powerful information on the amino acid sequences of these fragments [34].

It is thus apparent that there is a wealth of techniques available for the investigation of protein-protein interactions, but reinforces the need to analyse intact proteoforms. Processing of gene products into functional proteoforms impacts on the types of interactions these proteoforms may participate in. These global methodologies present a relatively inexpensive and rapid means to characterise protein-protein interactions, however it should be noted that no single technology is sufficient to confirm a binding event. Proteins that comprise part of a protein complex but that do not participate in direct binding to the bait represent a source of false positives. More targeted approaches such as ELISA [130], Surface Plasmon Resonance [131], and Thermophoresis [132] can be used to validate binding interactions.

## 8. The Detection of Low Abundance Proteoforms Using SDS-PAGE and Immunoblotting 

As mentioned above, the combination of 1D and 2D PAGE coupled with LC-MS/MS is an indispensable tool when investigating endoproteolytic processing as a PTM. However, there are often instances when even these techniques are not sensitive enough on their own to confirm the presence of low abundance proteoforms that are often beyond the limit of visualisation of 2D PAGE and are thus hidden, making subsequent LC-MS/MS analysis troublesome. For this, immunoblotting is an extremely powerful tool for the elucidation of these proteoforms. Western blotting involves the transferral of proteins from 1D or 2D-PAGE onto a semi-permeable membrane such as nitrocellulose or polyvinylidene fluoride (PVDF). These membranes can then be “probed” with either an antibody (immunoblotting [31]) or ligand (ligand blotting [32]) to investigate the presence/absence of a protein or binding interaction respectively. With the advancement of detection techniques such as enhanced chemiluminescence [133] and quantum dots [134], proteins can be detected down to the femtogram level. This allows for a greater flexibility when dealing with low sample yields and high cost reagents such as antibodies.

As an example of the technique, the P159 surface adhesin of *M. hyopneumoniae* was first characterised in 2006 and was shown to be cleaved into 3 distinct fragments [135]. Recombinant fragments spanning 4 distinct regions of the P159 preprotein were generated (F1–F4) for which corresponding polyclonal antisera was raised against. This allowed for immunoblots to be performed on whole cell lysates (WCLs) of *M. hyopneumoniae* to target the regions that antibodies were specific for [33]. 1D immunoblots of *M. hyopneumoniae* WCLs probed with F2 and F3 antisera revealed the dominant central fragment P110 in addition to multiple smaller mass fragments. Due to limitations in available technology at the time of the original publication, the sequences of these smaller and less abundant cleavage fragments could not be defined. In these blots, an intense band that resolves at ~75 kDa could be seen in both 1D immunoblots. A follow up study published in 2013 utilised 2D PAGE prior to immunoblotting and performed isoelectric focusing using separate 4–7 and 6–11 IPGs. This provided superior resolution of distinct proteoforms of P159, specifically the ~75 kDa fragment that was identified in 2006. This proteoform appeared to exist as a serious of spots that reacted intensely with F2 and F3 polyclonal antisera [33]. Interestingly, this proteoform appeared to undergo extensive modification due to the ‘spot training’ that occurred across both p*I* ranges. Given that this ~75 kDa fragment reacted with both F2 and F3 antisera, it would suggest that this proteoform is an endoproteolytic cleavage fragment of the central P110 proteoform. One would then expect a ~35 kDa fragment to exist and react with both F2 and F3 antisera and 1D and 2D immunoblots demonstrated such a fragment. At this point, true identification of these proteoforms via mass spectrometry is required. As was discussed above, affinity chromatography can be utilised for the enrichment of subproteomes such as surface proteins and was applied in this case, with the enriched surface proteins being separated by 2D PAGE and individual spots in gel digested and analysed by LC-MS/MS. From this, the approximate sequence of the 75 kDa fragment (termed P76) was identified. The cleavage site that generates P76 was also identified from a semi-tryptic peptide identified from an analogue of the ~35 kDa fragment mentioned above. This multifaceted analysis demonstrated that the P159 preprotein is extensively endoproteolytically cleaved into approximately 28 fragments demonstrating the need to move beyond the quantitation of gene products. Similar protocols were used successfully to characterise the multiple functional proteoforms derived from the P97 cilium adhesin [30,32], P216 [31], P146 [136] and other members of the P97 and P102 paralog families [137,138,139,140,141] of *M. hyopneumoniae* and to identify critical binding domains that target multiple host molecules. These studies highlight how a highly successful and economically significant pathogen with a reduced genome can expand the functional repertoire of its proteome by generating a complex array of functional proteoforms on the cell surface.

## 9. Top-Down Mass Spectrometry Methods for Proteoform Quantitation

As mentioned earlier and analogous to 2D-PAGE, the separation of the molecules of a unique proteoform from other proteoforms prior to ionisation by the mass spectrometer is critical for comprehensive proteome analysis. As the molecules of a single proteoform assume multiple different charge states during ionisation, if too many proteoforms are ionised simultaneously, the spectrum can become too complicated with charge states of different proteoforms intermingling. Fractionation is therefore critical to reduce the number of co-ionising proteoforms, with the complication that the isolated proteoform must be available in liquid to be ionised by electrospray. Liquid chromatography (LC) is a ubiquitous technology that can be coupled, in one form or another, to almost all forms of mass spectrometry through the use of appropriate volatile solvents, typically water, methanol and acetonitrile. Numerous variations of LC that have been employed in Top-Down analysis to try and separate proteins in such a way that unique proteoforms elute individually at a particular retention time. Multidimensional chromatography is often the first choice for complexity reduction, or more simply fractionating the sample by one type of chromatography (Strong Cation Exchange, for example) and subjecting the fractions produced to a different type of chromatography with orthogonal separation properties. Chen, et al. [142] utilised online hydrophobic interaction chromatography (HIC) followed by RP to improve chromatographic separation for complex protein mixes. HIC was chosen as it has a high sensitivity for conformational variation, does not denature proteins and is complementary to RP. The peaks from the chromatogram also resembled those of native mass spectra, confirming that native conditions had been maintained allowing for their intact analysis.

An alternative to RP chromatography, developed by Tran and Doucette [143] but applied to Top-Down Proteomics by the Kelleher lab, is the interestingly named GELFrEE (gel-eluted liquid fraction entrapment electrophoresis) system to fractionate samples prior to LC-MS/MS [143]. GELFrEE can use liquid phase IEF as the first dimension separation in a manner similar to a multi-compartment electrolyser, like the previously described ZoomIEFrunner, but most commonly the sample is solubilised in an SDS-based buffer and loaded into a horizontal tube of polyacrylamide gel (making the GELFrEE acronym somewhat misleading), separating the proteoforms by size into discreet ranges of increasing mass. These fractions are then subjected to an SDS removal step using either precipitation or membrane devices [144], followed by reverse phase chromatography which then separates the proteoforms by the orthogonal property of hydrophobicity using columns with beads of very large pore sizes (1000–4000 Å) to allow effective diffusion of the proteins into the pores and improved resolution and separation [145]. These three dimensions of separation provides a separation peak capacity that is similar to 2D-PAGE but with the need for 2–4 times the amount of sample ([7] supplementary data) . The ‘fourth’ dimension of separation is considered to be the mass spectrometer itself by making the assumption that 75 ‘peaks’ can be theoretically ‘fit’ in a 500–2000 *m*/*z* scan window ([7] supplementary data) however it is doubtful that 75 distinct proteoforms will be ionised together.

To achieve comprehensive Top-Down analysis of an entire proteome, the field must aim towards complete resolution of all proteoforms prior to ionisation into the mass spectrometer or excision from a gel. Neither chromatography, nor 2D-PAGE can currently offer this, however capillary electrophoresis (CE) may be capable of this resolution. CE, introduced in 1983 [146], separates molecules in open capillaries using electro-osmotic flow. Despite CE′s superior separation capacity, it has not seen routine analytical use for a number of reasons. To maximise separation efficiencies, nanolitre injection volumes of low protein concentration solution, 1–3 fold lower concentration than HPLC [147], are required to minimise band broadening and while techniques to ‘stack’ the analytes into a small volume in the capillary have been developed, such as electrophoretic sample stacking [148], field enhanced sample injection [149], and solid phase extraction [150], these methods do not completely fix the problem [151] nor are they in routine use. With the narrow elution peaks of a few seconds, the acquisition speed of the mass spectrometer was not sufficient for quantitative purposes, as not enough points across the peak could be acquired. Newer instruments with acquisition speeds of 100 Hz have solved this issue, even in cyclotron-based instruments, such as the Orbitrap, which can be programmed to increase acquisition times to improve mass resolution. The need to complete the electrical circuit in the capillary, while not interfering with electrospray ionisation where the capillary outlet is exposed to air is the greatest challenge of interfacing CE with MS [148]. This online interfacing [152] can be divided into two main groups, sheath-flow and sheathless. Sheath-flow, as the name suggests, employs a sheath fluid which the analytes must pass through prior to ionisation, a process that can cause sample dilution, although recent developments have minimised this [153,154]. Sheathless systems employ capillaries that have been made porous through hydrofluoric acid treatment, so that very small ions can pass through, completing the circuit [155] and this has recently been commercialised as the CESI-8000 interface. As CE-MS has already been shown to be capable of single cell metabolomics [156], steadily improving in Top-Down proteome coverage [152,157,158], and considering CE′s potential to resolve a complex proteome to single proteoforms prior to ionisation, it is likely that CE-MS will allow Top-Down proteomics to be performed at a greater depth and dynamic range than ever before.

Top-Down mass spectrometry of intact proteoforms in complex mixtures has been investigated extensively, although extensive purification to obtain a single, or at most a few, proteoforms at a single point in time is required, prior to ionisation. As with Bottom-Up methodologies, ionisation is most commonly performed by electrospray ionisation (ESI) coupled to reverse phase chromatography [159] or increasingly, capillary electrophoresis [153,157,160]. While other ionisation methods are available, ESI offers benefits that other methods do not. The multiply charged ions produced during electrospray means that instruments with small mass ranges of up to 3000–6000 *m*/*z* can accurately measure the mass of protein ions over 100,000 Da [26,161,162,163]. Each observed charge state can contain hundreds to thousands of molecules of the same proteoform with molecules differing by the number of 13-C atoms they contain. The resolution of these isotopes in a charge state makes calculation of proteoform mass more accurate but it requires the use of extremely high resolution mass spectrometers. Fourier Transform Ion Cyclotron Resonance Mass Spectrometry (FTICR-MS) is the most common form of high resolution instrument in Top-Down analysis due to its ability to resolve proteoforms to ~1 Da with a resolution or resolving power of >1,000,000 [164]. However, the requirement for liquid helium cooled magnets can result in a very high running cost (although manufacturers are addressing this issue), which has led researchers to another cyclotron-style instrument, the Orbitrap which uses the resonance of accelerated molecules to determine accurate mass [165,166]. Orbitraps are just as sensitive as FTICR MS with somewhat lower resolutions >500,000, however they also offer sub 1 Da distinction between intact proteoforms [167]. Very recently, there has been a report of using a 21-Tesla FTICR for proteome characterisation [168]. 82 h of instrument time was utilised to analyse 40 fractions of a single sample, identifying 684 unique protein entries or gene products and over 3200 proteoforms. This represents more than 50% of the highest coverage Top-Down MS dataset [20,35] but was acquired in 2% of the number of LC/MS/MS runs used in the previous studies. While this instrument provided impressive results, it only identified less than 5% of the predicted human proteome over 3.5 days and only one such instruments is available and thus it is of little use for the majority of laboratories until a commercial version of the instrument is available.

Similarly to shotgun LC/MS/MS methods for bottom up proteomics, advanced software is required in Top-Down MS methods to identify the gene product which best describes or ′matches′ to the MSMS spectrum produced by the fragmentation of the intact protein ion. The description of such tools and software is beyond the scope of this review, but the reader is directed to the Top-Down Proteomics Consortium′s software webpage which provides a comprehensive and up-to-date list of the available packages [169].

## 10. Ion Fragmentation and Selection

Direct, intact protein analysis via mass spectrometry has a number of limitations, foremost of which is the nature of ESI which will generate protein species with high charge states, potentially crowding relatively small *m*/*z* windows [170]. Attempts have been made to reduce the charge of molecules through the inclusion of acids, bases and gas phase ion/ion reactions, in an attempt to spread molecules out over a wider *m*/*z* range [171]. By using ion/ion interactions, ions can be ‘parked’ whereby ions are selected in an ion trap based on the decay of these charge states. As more molecules decay to the same charge state they are trapped thereby increasing signal of a particular *m*/*z* value [172]. An evolution of this technique in Top-Down analysis is termed targeted ion parking (TIPing) and it was pioneered by Campbell and Le Blanc [170] It uses a similar principle to the selected reaction monitoring (SRM) method for the quantitation of molecules i.e., preselected ions are targeted based on their expected charge states then ‘decayed’ to a single charge state before being accumulated for quantitation and measured. The advantage to such a technique relies on the fact that there is no Collision Induced Dissociation (CID) fragmentation of the molecules and therefore signals are not diluted by fragment data, nor are the parent ions lost in the fragmentation process. The overall benefit of this process is for the highly selected and filtered capturing of the same protein in varying charge states for accurate quantitation [173]. The application described in that work was quantitation of biotheraputic proteins and for an application requiring such a high level of specificity, the ability to only select the protein of interest, as well as its companion charge states, allows for a level of ion selection that would be beyond the capabilities of a normal SRM experiment.

It is important to keep in mind that each mature variant of a protein, including the multitude of PTM(s) which may occur at multiple locations within a protein, may be functionally distinct and should therefore be considered and quantitated separately. It is therefore necessary to select methods which are able to separate the signal of each of these protein species from each other, requiring the ability to detect distinct protein modifications. Top-Down mass spectrometry is now capable of characterising multiple functional PTMs in historically challenging protein species, such as transmembrane proteins [174]. In addition to mass spectrometry being used to measure the accurate mass of intact proteins, it is necessary to fragment these ions, as the fragmentation patterns can elucidate the amino acid sequence, as well as the specific location of any PTM(s) of the different proteoforms of the same amino acid sequence, which would otherwise be indistinguishable using exact mass measurements alone [167]. The choice of fragmentation method is also important, as it is common in conventional CID experiments to fragment or rearrange the bond between the PTM(s) and the protein, rather than simply cleave peptide bonds of the protein [175], thus losing the ability to identify and quantitate that proteoform. Electron Transfer Dissociation (ETD) fragmentation alone, or in combination with CID or the more recently developed High-energy Collisional Dissociation (HCD), is capable of retaining PTM(s) such as phosphorylation [176]. Thus Top-Down characterisation, down to proteoform resolution, is vital for true detection of changes in proteoform abundance, not just changes in the expression of a gene product which may potentially represent multiple proteoforms.

Relative ionisation efficiency of molecules in mass spectrometry is another consideration which may potentially affect the ability to perform relative quantitation of samples. Described by Smith et al. [177], the ionisation efficiencies of molecules can introduce bias with some proteoforms ionising better than others. It is important to note that the relative quantitation of the same proteoform in different samples relies on the assumption that the proteoform always has identical ionisation efficiency in the different samples. While all evidence indicates that this is true if the samples have been subjected to identical sample preparation steps that remove ′contaminants′ causing ion suppression, if it was not the case, data would be skewed towards assumptions that certain molecules are significantly up or down related in relation to others which would simply be untrue. Pesavento et al. [178] also investigated this by looking at proteoforms of the same H4 histone protein with the aim of determining the extent of the effect of ionisation efficiency on relative quantitation. They began by mixing equal ratios of acetylated isomers of H4 histone to see if the mass spectrometry could determine the correct relative quantity. In order to remove any potential conflicting variables, validation for the liquid chromatography was determined beforehand to ensure that elution ratios off the column were all equal; only minor variation (<5%) was found, meaning that all relative quantitation could be directly attributed to the instrumentation and not the chromatographic separation. It was found that higher ionisation efficiency and therefore a higher relative ratio was exhibited by proteins with more acetylations, despite the initial inclusion of equal quantities of 0, 1, 3 and 4 acetylated proteoforms. The characterisation of histone modifications has been a common use of Top-Down MS approaches [179,180].

## 11. Relative and Absolute Quantitation

While absolute quantitation using an internal standard to determine proteome changes is the ‘gold-standard’ to control for losses and variability which occur during sample handling, fractionation and ionisation, it is a difficult and expensive proposition for Top-Down Proteomics. It is unrealistic to manufacture a known quantity of stable isotope labelled analogues of all proteoforms, even if one considers producing recombinant proteins in bacterial or eukaryotic systems grown in a source of heavy carbon or nitrogen. Thus, methods of relative quantitation are employed, such as the DiGE method mentioned previously. One method that has been employed is a ’label free’ mass spectrometric method of relative quantitation of complex bacterial lysates using the proteomic equivalent of ’house-keeping genes’ i.e., proteins that do not change, quantitatively, in a significant way between samples. This method was proposed by Williams et al. [181] and focused on identifying two proteins that exhibited similar levels of protein expression between different strains of *E. coli*. Once these proteins had been identified they were used as a scale to determine relative up and down regulation of other proteins. This methodology is very similar to other differential display workflows (mentioned above in 2D gels) as it provides a direct comparison between 2 different biological samples, using the total amount protein as the reference. The label free approach has also been explored in recent work by the Kelleher lab resulting in an analysis pipeline that can be applied to complex proteome samples [182].

Other MS-based methods of relative quantitation add unique mass ′tags′ to each sample to be measured by the mass spectrometer. The labels are isobaric which enables signal from each sample to be detected at the same point in time in the mass spectrometer. These methods include isobaric Tagging for Relative and Absolute Quantification (iTRAQ) [183,184], Tandem Mass Tags (TMT) [185], multiplexed stable isotope dimethyl labelling [186] and Isotope Coded Affinity Tags (ICAT) [187]. The iTRAQ method, for example, employs a multiplexed stable isotope label of all proteins in either four or eight different biological samples, allowing simultaneous relative quantitative analysis of protein abundance. Similarly to DiGE, the proteins are mixed in equal ratios and then subjected to fractionation, usually by reversed phase chromatography, prior to mass spectrometry. Fragmentation releases reporter ions for each sample which is then computed for relative intensity between samples, inferring abundance changes for each proteoform. In replicates, it is recommended to switch the label for the control and treated samples to ensure there is no technical variability or bias attributed to the different labels. While these methods were designed with Bottom-Up strategies in mind, the TMT labelling method has been successfully applied to Top-Down, intact protein mass spectrometry using multiplexed model proteins in a LTQ-Orbitrap Velos which demonstrated that the technique could accurately quantify the expected relative abundances of proteoforms [188]. Caution must be exercised during the labelling steps because the labelling chemicals themselves are poorly soluble in aqueous solutions and require solvents such as 100% ethanol to be solubilised. Adding too high a concentration of any organic solvent to a protein sample can cause protein precipitation and potential loss of sample.

These chemical protein labelling methods rely on the complete labelling of targeted residues, as incomplete labelling results in the creation of either multiple chromatographic peaks or a range of charge states with different masses due to differing numbers of labels on different molecules of the same proteoform, reducing and separating the signal of each unique proteoform to be quantitated. Complete labelling may or may not be achieved depending on the buffers required to solubilise the protein samples and the degree of steric hindrance inherent to the proteins of interest. As with many proteomic methodologies, the method suffers most from samples with high dynamic range and requires careful controls to ensure no bias is introduced in final analysis and biological interpretation [189]. However, Top-Down strategies alleviate some of the issues noted with peptide bias in Bottom-Up mass spectrometry when quantitating proteoforms [190].

An alternative to chemical protein labelling quantitation techniques, is the metabolic labelling of proteins, which avoids the issues of poor and/or variable protein labelling efficiencies. These methods introduce stable isotopic elements such as purified ^15^N or amino acids which are incorporated by live cells during protein turnover throughout cell growth. To ensure completeness, the culture is usually grown for multiple generations. There are a number of choices of metabolic label, each with their own limitations, many of which have been applied in model organisms (reviewed in Gouw et al., [191]).

The most widely used metabolic labelling technique is Stable Isotope Labelling of Amino acids in Cell culture (SILAC), developed by the group of Matthias Mann [192,193]. SILAC involves supplementing cell culture media with stable isotope versions of amino acids, typically ^13^C_6_ or ^13^C_6_^15^N_5_ arginine and/or lysine. When cells are grown in this modified medium, the ′heavy′ amino acids are fully incorporated into proteins during the cell′s normal protein synthesis pathways after five to ten cell divisions. A second control population of cells is grown with a supplement of unlabelled amino acids (’light label’). As with other labelling techniques the light and heavy labelled amino acids are usually switched. The observed ion intensity ratio between the resulting peptides provides the relative differential expression of proteins in response to the changed growth conditions. In addition to the benefit of complete of incorporation of the label into proteins, this method allows mixing of the sample to be compared using equal cell counts or cell weights prior to cellular lysis and protein extraction. This avoids technical variations caused by variations in sample-to-sample extraction efficiency, inaccuracy in protein measurement, as well as errors in mixing equal quantities of total protein.

The SILAC method has been evaluated using expressed Grb2 signalling protein, which found the mass difference between metabolically labelled and unlabelled forms more predictable in contrast to the stochastic incorporation of ^15^N labelling, simplifying Top-Down intact protein mass spectrometry [194]. In an analysis of ^15^N labelled proteins from *S. cerevisiae* grown aerobically vs. anaerobically, 231 paired proteoforms with molecular weights between 14–35 kDa were detected by intact protein mass spectrometry and used to compare protein abundance changes. However, fragmentation and protein identification was not possible for all of these proteins [159]. By comparison, 659 proteoform pairs of SILAC labelled of *Aspergillus flavus* were detected by intact protein mass spectrometry with 22 confident identifications [195].

Absolute quantitation has also been carried out using the Protein Standard Absolute Quantitation (PSAQ) methodology, which shares similarity to the Absolute Quantitation (AQUA) peptide strategy where the well-established isotope dilution principle of spiking in isotopically labelled peptides of known quantity is utilised. PSAQ uses recombinant fusion proteins, which can be produced using cell-free synthesis, that most often contain ^13^C_6_, ^15^N_2_ Lysine and ^13^C_6_, ^15^N_4_ Arginine with a cleavable hexahistidine tag for purification [196,197]. The addition of only ′heavy′ lysine and arginine was to allow the intact standard protein to be spiked into the sample at a known amount at an early point in sample preparation and the sample digested to peptides for shotgun LC/MS/MS analysis. This approach was found to have greater quantitative accuracy when compared to AQUA and compatible with SDS-PAGE and protein capture techniques [197,198]. Being an intact protein standard, PSAQ would also compatible with Top-Down approaches. In Top-Down MS, isotope dilution has been used to measure insulin levels [199,200], and while not yet reported, there is no reason that PSAQ could not be used in 2D-PAGE where the standard would co-resolve with its unlabelled homologue and the label of the peptides liberated by in gel digestion of the protein can be used to absolutely quantitate the amount of peptide and therefore protein present by LC/MS/MS. The drawback of this technique is the time and resources required to make the isotopically labelled proteins, which would explain why there are few reports of it being used for Top-Down quantification.

## 12. Conclusions

The study of the proteome is essential to understanding the genotype phenotype nexus. Despite significant advances in the sensitivity and speed attained by modern mass spectrometers, advances in the study of proteoforms and protein complexes remains in its infancy. New researchers to the proteomics field can be forgiven for thinking that the field is focused on cataloguing the abundance of gene products through Bottom-Up techniques as a measure of proteome changes and thus changes in phenotype. This ignores the fact that it is the final protein product, or proteoform, that is the functional unit that defines a cell′s phenotype from a proteome viewpoint. Since Patrick O′Farrell′s 1975 publication of 2D-PAGE, the number of intact proteoforms able to be detected and identified has not substantially increased, with an approximate doubling of observable spots on 2D-PAGE and a similar number identified by Top-Down MS. The complete and comprehensive analysis of the proteome using Top-Down approaches is still beyond our reach. What this article tries to point out is that researchers are faced with stark choices when commencing the analysis of a cell′s or tissue′s proteome, choices that are determined by sample availability and equipment availability rather than technical difficulty. It is the author′s firm opinion that, provided equipment is available, there are a great deal of resources available in the published literature to guide even the most inexperienced researcher in the most appropriate techniques in sample preparation and fractionation to use in proteomics analysis. The ‘myths’ of 2D-PAGE have been dispelled in numerous articles and while LC-MS/MS of intact proteoforms is a daunting prospect compared to peptides, the problems encountered are nearly always due to poor sample preparation, as they are for 2D-PAGE, causing problems in reversed phase chromatography and ionisation.

## Figures and Tables

**Table 1:** Summary of the commonly used techniques described in this review, including brief comments on their advantages and disadvantages.

Technique	Advantages	Disadvantages	Reference
Shotgun LC/MS/MS	High proteome coverage.	Proteoform information disconnected from measured peptides unless particular peptide detected	[8,10,16,17,18]
1D-PAGE/Shotgun LC/MS/MS	Proteoform size preserved allowing proteolytic cleavage of parent protein to be inferred. High proteome coverage. SDS can be used to solubilise proteins.	Proteoform information disconnected from measured peptides unless particular peptide detected.	[32,33,34]
2D-PAGE	High resolution separation of intact proteoforms. Parallel processing of multiple samples. Robust quantitation of proteoforms	High amount of sample required compared to shotgun LC/MS/MS. Perception of high technical difficulty. Low proteome coverage compared to LC/MS/MS	[21]
GelFREE LC/MS/MS	High accuracy measurement of proteoform mass that can infer nature of PTMs or proteolytic cleavage.	Low proteome coverage compared to LC/MS/MS. Cannot parallel process multiple samples. Enormous amount of MS acquisition time required for one sample resulting in low throughput.	[20,35]
Native PAGE	Maintains biological context of protein-protein interaction	Sample handling needs to be controlled for temperature, pH and physical movement. Transient interactions can be easily lost if these parameters are not maintained.	[36,37,38]
Ligand blotting	Supportive orthogonal method to confirm interactions between 2 or more molecules	Titration of ligand to binding partner requires optimisation as in antibody western blot systems	[31]
Bait-prey affinity isolation	Allows for a robust labelled capture-based technique for interacting proteins	Precipitation of proteins in sample preparation can preclude them from the method. False-positive interactions can occur with binding sites of proteins folding changes in altered buffering conditions.	[31,34]

## References

[1] Smith L.M., Kelleher N.L. (2013). Consortium for Top Down, P. Proteoform: A single term describing protein complexity. Nat. Methods.

[2] Kelleher N.L., Lin H.Y., Valaskovic G.A., Aaserud D.J., Fridriksson E.K., McLafferty F.W. (1999). Top down versus bottom up protein characterization by tandem high-resolution mass spectrometry. J. Am. Chem. Soc..

[3] Anderson S. (1981). Shotgun DNA sequencing using cloned dnase i-generated fragments. Nucleic Acids Res..

[4] Yates J.R. (1998). Mass spectrometry and the age of the proteome. J. Mass Spectrom..

[5] Kelleher N.L. (2004). Top-down proteomics. Anal. Chem..

[6] Pandey A., Mann M. (2000). Proteomics to study genes and genomes. Nature.

[7] Tran J.C., Zamdborg L., Ahlf D.R., Lee J.E., Catherman A.D., Durbin K.R., Tipton J.D., Vellaichamy A., Kellie J.F., Li M. (2011). Mapping intact protein isoforms in discovery mode using top-down proteomics. Nature.

[8] Thakur S.S., Geiger T., Chatterjee B., Bandilla P., Frohlich F., Cox J., Mann M. (2011). Deep and highly sensitive proteome coverage by lc-ms/ms without prefractionation. Mol. Cell. Proteom..

[9] Hosp F., Scheltema R.A., Eberl H.C., Kulak N.A., Keilhauer E.C., Mayr K., Mann M. (2015). A double-barrel liquid chromatography-tandem mass spectrometry (lc-ms/ms) system to quantify 96 interactomes per day. Mol. Cell. Proteom..

[10] Grassl N., Kulak N.A., Pichler G., Geyer P.E., Jung J., Schubert S., Sinitcyn P., Cox J., Mann M. (2016). Ultra-deep and quantitative saliva proteome reveals dynamics of the oral microbiome. Genome Med..

[11] Richards A.L., Hebert A.S., Ulbrich A., Bailey D.J., Coughlin E.E., Westphall M.S., Coon J.J. (2015). One-hour proteome analysis in yeast. Nat. Protoc..

[12] Taylor C.F., Paton N.W., Lilley K.S., Binz P.A., Julian R.K., Jones A.R., Zhu W., Apweiler R., Aebersold R., Deutsch E.W. (2007). The minimum information about a proteomics experiment (miape). Nat. Biotechnol..

[13] Lluch-Senar M., Delgado J., Chen W.H., Llorens-Rico V., O'Reilly F.J., Wodke J.A., Unal E.B., Yus E., Martinez S., Nichols R.J. (2015). Defining a minimal cell: Essentiality of small orfs and ncrnas in a genome-reduced bacterium. Mol. Syst. Biol..

[14] Rabilloud T., Lescuyer P. (2014). The proteomic to biology inference, a frequently overlooked concern in the interpretation of proteomic data: A plea for functional validation. Proteomics.

[15] Nesvizhskii A.I., Aebersold R. (2005). Interpretation of shotgun proteomic data: The protein inference problem. Mol. Cell. Proteom..

[16] Richards A.L., Merrill A.E., Coon J.J. (2015). Proteome sequencing goes deep. Curr. Opin. Chem. Biol..

[17] Wilhelm M., Schlegl J., Hahne H., Moghaddas Gholami A., Lieberenz M., Savitski M.M., Ziegler E., Butzmann L., Gessulat S., Marx H. (2014). Mass-spectrometry-based draft of the human proteome. Nature.

[18] Kim M.S., Pinto S.M., Getnet D., Nirujogi R.S., Manda S.S., Chaerkady R., Madugundu A.K., Kelkar D.S., Isserlin R., Jain S. (2014). A draft map of the human proteome. Nature.

[19] Ezkurdia I., Vazquez J., Valencia A., Tress M. (2014). Analyzing the first drafts of the human proteome. J. Proteome Res..

[20] Durbin K.R., Fornelli L., Fellers R.T., Doubleday P.F., Narita M., Kelleher N.L. (2016). Quantitation and identification of thousands of human proteoforms below 30 kda. J. Proteome Res..

[21] Wright E.P., Partridge M.A., Padula M.P., Gauci V.J., Malladi C.S., Coorssen J.R. (2014). Top-down proteomics: Enhancing 2d gel electrophoresis from tissue processing to high-sensitivity protein detection. Proteomics.

[22] Kuljanin M., Dieters-Castator D.Z., Hess D.A., Postovit L.M., Lajoie G.A. (2016). Comparison of sample preparation techniques for large scale proteomics. Proteomics.

[23] Issaq H.J., Conrads T.P., Janini G.M., Veenstra T.D. (2002). Methods for fractionation, separation and profiling of proteins and peptides. Electrophoresis.

[24] Pieper R., Gatlin C.L., Makusky A.J., Russo P.S., Schatz C.R., Miller S.S., Su Q., McGrath A.M., Estock M.A., Parmar P.P. (2003). The human serum proteome: Display of nearly 3700 chromatographically separated protein spots on two-dimensional electrophoresis gels and identification of 325 distinct proteins. Proteomics.

[25] Grandori R. (2003). Origin of the conformation dependence of protein charge-state distributions in electrospray ionization mass spectrometry. J. Mass Spectrom..

[26] Marshall A.G., Senko M.W., Li W., Li M., Dillon S., Guan S., Logan T.M. (1997). Protein molecular mass to 1 da by 13c, 15n double-depletion and ft-icr mass spectrometry. J. Am. Chem. Soc..

[27] Lomeli S.H., Yin S., Ogorzalek Loo R.R., Loo J.A. (2009). Increasing charge while preserving noncovalent protein complexes for esi-ms. J. Am. Soc. Mass Spectrom..

[28] Sterling H.J., Daly M.P., Feld G.K., Thoren K.L., Kintzer A.F., Krantz B.A., Williams E.R. (2010). Effects of supercharging reagents on noncovalent complex structure in electrospray ionization from aqueous solutions. J. Am. Soc. Mass Spectrom..

[29] Zenaidee M.A., Donald W.A. (2015). Extremely supercharged proteins in mass spectrometry: Profiling the ph of electrospray generated droplets, narrowing charge state distributions, and increasing ion fragmentation. Analyst.

[30] Djordjevic S.P., Cordwell S.J., Djordjevic M.A., Wilton J., Minion F.C. (2004). Proteolytic processing of the mycoplasma hyopneumoniae cilium adhesin. Infect. Immun..

[31] Tacchi J.L., Raymond B.B., Jarocki V.M., Berry I.J., Padula M.P., Djordjevic S.P. (2014). Cilium adhesin p216 (mhj_0493) is a target of ectodomain shedding and aminopeptidase activity on the surface of mycoplasma hyopneumoniae. J. Proteome Res..

[32] Raymond B.B., Jenkins C., Seymour L.M., Tacchi J.L., Widjaja M., Jarocki V.M., Deutscher A.T., Turnbull L., Whitchurch C.B., Padula M.P. (2015). Proteolytic processing of the cilium adhesin mhj_0194 (p123j ) in mycoplasma hyopneumoniae generates a functionally diverse array of cleavage fragments that bind multiple host molecules. Cell Microbiol..

[33] Raymond B.B., Tacchi J.L., Jarocki V.M., Minion F.C., Padula M.P., Djordjevic S.P. (2013). P159 from mycoplasma hyopneumoniae binds porcine cilia and heparin and is cleaved in a manner akin to ectodomain shedding. J. Proteome Res..

[34] Tacchi J.L., Raymond B.B., Haynes P.A., Berry I.J., Widjaja M., Bogema D.R., Woolley L.K., Jenkins C., Minion F.C., Padula M.P. (2016). Post-translational processing targets functionally diverse proteins in mycoplasma hyopneumoniae. Open. Biol..

[35] Catherman A.D., Durbin K.R., Ahlf D.R., Early B.P., Fellers R.T., Tran J.C., Thomas P.M., Kelleher N.L. (2013). Large-scale top-down proteomics of the human proteome: Membrane proteins, mitochondria, and senescence. Mol. Cell. Proteomics.

[36] Wittig I., Braun H.P., Schagger H. (2006). Blue native page. Nat. Protoc..

[37] Wittig I., Schagger H. (2009). Native electrophoretic techniques to identify protein-protein interactions. Proteomics.

[38] Zickermann V., Wumaier Z., Wrzesniewska B., Hunte C., Schagger H. (2010). Native immunoblotting of blue native gels to identify conformation-specific antibodies. Proteomics.

[39] O’Farrell P.H. (1975). High resolution two-dimensional electrophoresis of proteins. J. Biol. Chem..

[40] Westermeier R. (2014). Looking at proteins from two dimensions: A review on five decades of 2d electrophoresis. Arch. Physiol. Biochem..

[41] O’Farrell P.Z., Goodman H.M., O’Farrell P.H. (1977). High resolution two-dimensional electrophoresis of basic as well as acidic proteins. Cell.

[42] Gorg A., Postel W., Westermeier R. (1978). Ultrathin-layer isoelectric focusing in polyacrylamide gels on cellophane. Anal. Biochem..

[43] Bjellqvist B., Ek K., Righetti P.G., Gianazza E., Gorg A., Westermeier R., Postel W. (1982). Isoelectric focusing in immobilized ph gradients: Principle, methodology and some applications. J. Biochem. Biophys. Methods.

[44] Churchward M.A., Butt R.H., Lang J.C., Hsu K.K., Coorssen J.R. (2005). Enhanced detergent extraction for analysis of membrane proteomes by two-dimensional gel electrophoresis. Proteome Sci..

[45] Butt R.H., Pfeifer T.A., Delaney A., Grigliatti T.A., Tetzlaff W.G., Coorssen J.R. (2007). Enabling coupled quantitative genomics and proteomics analyses from rat spinal cord samples. Mol. Cell. Proteom..

[46] Molloy M.P., Herbert B.R., Williams K.L., Gooley A.A. (1999). Extraction of escherichia coli proteins with organic solvents prior to two-dimensional electrophoresis. Electrophoresis.

[47] Herbert B.R., Harry J.L., Packer N.H., Gooley A.A., Pedersen S.K., Williams K.L. (2001). What place for polyacrylamide in proteomics?. Trends Biotechnol..

[48] Herbert B.R., Grinyer J., McCarthy J.T., Isaacs M., Harry E.J., Nevalainen H., Traini M.D., Hunt S., Schulz B., Laver M. (2006). Improved 2-de of microorganisms after acidic extraction. Electrophoresis.

[49] Rabilloud T. (2010). Variations on a theme: Changes to electrophoretic separations that can make a difference. J. Proteomics.

[50] Rabilloud T., Lelong C. (2011). Two-dimensional gel electrophoresis in proteomics: A tutorial. J. Proteomics.

[51] Peaks amino acid mass table. http://www.bioinfor.com/wp-content/uploads/2017/01/2015-massref-web.pdf.

[52] Wu X., Xiong E., Wang W., Scali M., Cresti M. (2014). Universal sample preparation method integrating trichloroacetic acid/acetone precipitation with phenol extraction for crop proteomic analysis. Nat. Protoc..

[53] Butt R.H., Coorssen J.R. (2006). Pre-extraction sample handling by automated frozen disruption significantly improves subsequent proteomic analyses. J. Proteome Res..

[54] Molloy M.P., Herbert B.R., Walsh B.J., Tyler M.I., Traini M., Sanchez J.C., Hochstrasser D.F., Williams K.L., Gooley A.A. (1998). Extraction of membrane proteins by differential solubilization for separation using two-dimensional gel electrophoresis. Electrophoresis.

[55] Blisnick T., Morales-Betoulle M.E., Vuillard L., Rabilloud T., Braun Breton C. (1998). Non-detergent sulphobetaines enhance the recovery of membrane and/or cytoskeleton-associated proteins and active proteases from erythrocytes infected by plasmodium falciparum. Eur. J. Biochem..

[56] Chevallet M., Santoni V., Poinas A., Rouquie D., Fuchs A., Kieffer S., Rossignol M., Lunardi J., Garin J., Rabilloud T. (1998). New zwitterionic detergents improve the analysis of membrane proteins by two-dimensional electrophoresis. Electrophoresis.

[57] Goldberg M.E., Expert-Bezancon N., Vuillard L., Rabilloud T. (1996). Non-detergent sulphobetaines: A new class of molecules that facilitate in vitro protein renaturation. Fold Des..

[58] Rabilloud T., Gianazza E., Catto N., Righetti P.G. (1990). Amidosulfobetaines, a family of detergents with improved solubilization properties: Application for isoelectric focusing under denaturing conditions. Anal. Biochem..

[59] Chertov O., Biragyn A., Kwak L.W., Simpson J.T., Boronina T., Hoang V.M., Prieto D.A., Conrads T.P., Veenstra T.D., Fisher R.J. (2004). Organic solvent extraction of proteins and peptides from serum as an effective sample preparation for detection and identification of biomarkers by mass spectrometry. Proteomics.

[60] Herbert B.R., Molloy M.P., Gooley A.A., Walsh B.J., Bryson W.G., Williams K.L. (1998). Improved protein solubility in two-dimensional electrophoresis using tributyl phosphine as reducing agent. Electrophoresis.

[61] Gordon J.A., Jencks W.P. (1963). The relationship of structure to the effectiveness of denaturing agents for proteins. Biochemistry.

[62] Gronow M., Griffiths G. (1971). Rapid isolation and separation of the non-histone proteins of rat liver nuclei. FEBS Lett..

[63] Rabilloud T., Adessi C., Giraudel A., Lunardi J. (1997). Improvement of the solubilization of proteins in two-dimensional electrophoresis with immobilized ph gradients. Electrophoresis.

[64] Rabilloud T. (1998). Use of thiourea to increase the solubility of membrane proteins in two-dimensional electrophoresis. Electrophoresis.

[65] Rabilloud T. (1999). Solubilization of proteins in 2-d electrophoresis. An outline. Methods Mol. Biol..

[66] Zhou R., Li J., Hua L., Yang Z., Berne B.J. (2011). Comment on “urea-mediated protein denaturation: A consensus view”. J. Phys. Chem. B.

[67] Perdew G.H., Schaup H.W., Selivonchick D.P. (1983). The use of a zwitterionic detergent in two-dimensional gel electrophoresis of trout liver microsomes. Anal. Biochem..

[68] Gianazza E., Rabilloud T., Quaglia L., Caccia P., Astrua-Testori S., Osio L., Grazioli G., Righetti P.G. (1987). Additives for immobilized ph gradient two-dimensional separation of particulate material: Comparison between commercial and new synthetic detergents. Anal. Biochem..

[69] Rabilloud T., Blisnick T., Heller M., Luche S., Aebersold R., Lunardi J., Braun-Breton C. (1999). Analysis of membrane proteins by two-dimensional electrophoresis: Comparison of the proteins extracted from normal or plasmodium falciparum-infected erythrocyte ghosts. Electrophoresis.

[70] Luche S., Santoni V., Rabilloud T. (2003). Evaluation of nonionic and zwitterionic detergents as membrane protein solubilizers in two-dimensional electrophoresis. Proteomics.

[71] Herbert B., Hopwood F., Oxley D., McCarthy J., Laver M., Grinyer J., Goodall A., Williams K., Castagna A., Righetti P.G. (2003). Beta-elimination: An unexpected artefact in proteome analysis. Proteomics.

[72] Luche S., Diemer H., Tastet C., Chevallet M., Van Dorsselaer A., Leize-Wagner E., Rabilloud T. (2004). About thiol derivatization and resolution of basic proteins in two-dimensional electrophoresis. Proteomics.

[73] Sechi S., Chait B.T. (1998). Modification of cysteine residues by alkylation. A tool in peptide mapping and protein identification. Anal. Chem..

[74] Aitken A., Learmonth M., Walker J.M. (2002). Carboxymethylation of cysteine using iodoacetamide/ iodoacetic acid. The Protein Protocols Handbook.

[75] Patterson S.D., Aebersold R. (1995). Mass spectrometric approaches for the identification of gel-separated proteins. Electrophoresis.

[76] Patterson S.D. (1995). Matrix-assisted laser-desorption/ionization mass spectrometric approaches for the identification of gel-separated proteins in the 5–50 pmol range. Electrophoresis.

[77] McCarthy J., Hopwood F., Oxley D., Laver M., Castagna A., Righetti P.G., Williams K., Herbert B. (2003). Carbamylation of proteins in 2-d electrophoresis—Myth or reality?. J. Proteome Res..

[78] Anderson N.L. (2002). The human plasma proteome: History, character, and diagnostic prospects. Mol. Cell. Proteom..

[79] Righetti P.G., Castagna A., Antonioli P., Boschetti E. (2005). Prefractionation techniques in proteome analysis: The mining tools of the third millennium. Electrophoresis.

[80] Righetti P.G., Castagna A., Herbert B., Reymond F., Rossier J.S. (2003). Prefractionation techniques in proteome analysis. Proteomics.

[81] Zubarev R.A. (2013). The challenge of the proteome dynamic range and its implications for in-depth proteomics. Proteomics.

[82] Pasquali C., Fialka I., Huber L.A. (1997). Preparative two-dimensional gel electrophoresis of membrane proteins. Electrophoresis.

[83] Padula M.P. (2009). The Development of Proteomic Techniques to Study the Australian Paralysis Tick, Ixodes Holocyclus: The Application of Proteomic Technology to an Organism with Poor Bioinformatic Information.

[84] D’Amici G.M., Timperio A.M., Zolla L. (2008). Coupling of native liquid phase isoelectrofocusing and blue native polyacrylamide gel electrophoresis: A potent tool for native membrane multiprotein complex separation. J. Proteome Res..

[85] Ayala A., Parrado J., Machado A. (1998). Use of rotofor preparative isoelectrofocusing cell in protein purification procedure. Appl. Biochem. Biotechnol..

[86] Zuo X., Speicher D.W. (2000). A method for global analysis of complex proteomes using sample prefractionation by solution isoelectrofocusing prior to two-dimensional electrophoresis. Anal. Biochem..

[87] Zuo X., Speicher D.W. (2002). Comprehensive analysis of complex proteomes using microscale solution isoelectrofocusing prior to narrow ph range two-dimensional electrophoresis. Proteomics.

[88] Ros A., Faupel M., Mees H., Oostrum J., Ferrigno R., Reymond F., Michel P., Rossier J.S., Girault H.H. (2002). Protein purification by off-gel electrophoresis. Proteomics.

[89] Heller M., Michel P.E., Morier P., Crettaz D., Wenz C., Tissot J.D., Reymond F., Rossier J.S. (2005). Two-stage off-gel isoelectric focusing: Protein followed by peptide fractionation and application to proteome analysis of human plasma. Electrophoresis.

[90] Bogema D.R., Scott N.E., Padula M.P., Tacchi J.L., Raymond B.B., Jenkins C., Cordwell S.J., Minion F.C., Walker M.J., Djordjevic S.P. (2011). Sequence ttkf downward arrow qe defines the site of proteolytic cleavage in mhp683 protein, a novel glycosaminoglycan and cilium adhesin of mycoplasma hyopneumoniae. J. Biol. Chem..

[91] Edman P., Begg G. (1967). A protein sequenator. Eur. J. Biochem..

[92] Edman P. (1949). A method for the determination of amino acid sequence in peptides. Arch. Biochem..

[93] Gevaert K., Goethals M., Martens L., Van Damme J., Staes A., Thomas G.R., Vandekerckhove J. (2003). Exploring proteomes and analyzing protein processing by mass spectrometric identification of sorted n-terminal peptides. Nat. Biotechnol..

[94] Percent semi-tryptic. http://massqc.proteomesoftware.com/help/metrics/percent_semi_tryptic.

[95] Kleifeld O., Doucet A., Prudova A., auf dem Keller U., Gioia M., Kizhakkedathu J.N., Overall C.M. (2011). Identifying and quantifying proteolytic events and the natural n terminome by terminal amine isotopic labeling of substrates. Nat. Protoc..

[96] Rabilloud T. (2009). Membrane proteins and proteomics: Love is possible, but so difficult. Electrophoresis.

[97] Bononi A., Agnoletto C., De Marchi E., Marchi S., Patergnani S., Bonora M., Giorgi C., Missiroli S., Poletti F., Rimessi A. (2011). Protein kinases and phosphatases in the control of cell fate. Enzyme Res..

[98] Reisinger V., Eichacker L.A. (2008). Solubilization of membrane protein complexes for blue native page. J. Proteomics.

[99] Krause F. (2006). Detection and analysis of protein-protein interactions in organellar and prokaryotic proteomes by native gel electrophoresis: (membrane) Protein complexes and supercomplexes. Electrophoresis.

[100] Szilagyi A., Grimm V., Arakaki A.K., Skolnick J. (2005). Prediction of physical protein-protein interactions. Phys. Biol..

[101] Le Maire M., Champeil P., Møller J.V. (2000). Interaction of membrane proteins and lipids with solubilizing detergents. Biochim. Biophys. Acta (BBA) Biomembr..

[102] Fiala G.J., Schamel W.W.A., Blumenthal B. (2011). Blue native polyacrylamide gel electrophoresis (bn-page) for analysis of multiprotein complexes from cellular lysates. J. Vis. Exp. JoVE.

[103] Zheng J., Wei C., Zhao L., Liu L., Leng W., Li W., Jin Q. (2011). Combining blue native polyacrylamide gel electrophoresis with liquid chromatography tandem mass spectrometry as an effective strategy for analyzing potential membrane protein complexes of mycobacterium bovis bacillus calmette-guérin. BMC Genom..

[104] Robinson M.W., Buchtmann K.A., Jenkins C., Tacchi J.L., Raymond B.B., To J., Roy Chowdhury P., Woolley L.K., Labbate M., Turnbull L. (2013). Mhj_0125 is an m42 glutamyl aminopeptidase that moonlights as a multifunctional adhesin on the surface of mycoplasma hyopneumoniae. Open Biol..

[105] Dudkina N.V., Eubel H., Keegstra W., Boekema E.J., Braun H.P. (2005). Structure of a mitochondrial supercomplex formed by respiratory-chain complexes i and iii. Proc. Natl. Acad. Sci. USA.

[106] Sokolova L., Wittig I., Barth H.D., Schagger H., Brutschy B., Brandt U. (2010). Laser-induced liquid bead ion desorption-ms of protein complexes from blue-native gels, a sensitive top-down proteomic approach. Proteomics.

[107] Gault J., Donlan J.A., Liko I., Hopper J.T., Gupta K., Housden N.G., Struwe W.B., Marty M.T., Mize T., Bechara C. (2016). High-resolution mass spectrometry of small molecules bound to membrane proteins. Nat. Methods.

[108] Hopper J.T., Robinson C.V. (2014). Mass spectrometry quantifies protein interactions—From molecular chaperones to membrane porins. Angew. Chem. Int. Ed. Engl..

[109] Schmidt C., Robinson C.V. (2014). A comparative cross-linking strategy to probe conformational changes in protein complexes. Nat. Protoc..

[110] Towbin H., Staehelin T., Gordon J. (1979). Electrophoretic transfer of proteins from polyacrylamide gels to nitrocellulose sheets: Procedure and some applications. Proc. Natl. Acad. Sci. USA.

[111] Smith P.K., Krohn R.I., Hermanson G.T., Mallia A.K., Gartner F.H., Provenzano M.D., Fujimoto E.K., Goeke N.M., Olson B.J., Klenk D.C. (1985). Measurement of protein using bicinchoninic acid. Anal. Biochem..

[112] Lowry O.H., Rosebrough N.J., Farr A.L., Randall R.J. (1951). Protein measurement with the folin phenol reagent. J. Biol. Chem..

[113] Bradford M.M. (1976). A rapid and sensitive method for the quantitation of microgram quantities of protein utilising the principle of protein-dye binding. Anal. Biochem..

[114] Unlu M., Morgan M.E., Minden J.S. (1997). Difference gel electrophoresis: A single gel method for detecting changes in protein extracts. Electrophoresis.

[115] Marouga R., David S., Hawkins E. (2005). The development of the dige system: 2d fluorescence difference gel analysis technology. Anal. Bioanal. Chem..

[116] Keeping A.J., Collins R.A. (2011). Data variance and statistical significance in 2d-gel electrophoresis and dige experiments: Comparison of the effects of normalization methods. J. Proteome Res..

[117] Kondo T., Hirohashi S. (2006). Application of highly sensitive fluorescent dyes (cydye dige fluor saturation dyes) to laser microdissection and two-dimensional difference gel electrophoresis (2d-dige) for cancer proteomics. Nat. Protoc..

[118] Sriharshan A., Boldt K., Sarioglu H., Barjaktarovic Z., Azimzadeh O., Hieber L., Zitzelsberger H., Ueffing M., Atkinson M.J., Tapio S. (2012). Proteomic analysis by silac and 2d-dige reveals radiation-induced endothelial response: Four key pathways. J. Proteom..

[119] Phizicky E.M., Fields S. (1995). Protein-protein interactions: Methods for detection and analysis. Microbiol. Rev..

[120] De Gunzburg J., Riehl R., Weinberg R.A. (1989). Identification of a protein associated with p21ras by chemical crosslinking. Proc. Natl. Acad. Sci. USA.

[121] Fields S., Song O. (1989). A novel genetic system to detect protein-protein interactions. Nature.

[122] Miller K.G., Alberts B.M. (1989). F-actin affinity chromatography: Technique for isolating previously unidentified actin-binding proteins. Proc. Natl. Acad. Sci. USA.

[123] Widjaja M., Berry I., Pont E., Padula M., Djordjevic S. (2015). P40 and p90 from mpn142 are targets of multiple processing events on the surface of mycoplasma pneumoniae. Proteomes.

[124] Schiapparelli L.M., McClatchy D.B., Liu H.H., Sharma P., Yates J.R., Cline H.T. (2014). Direct detection of biotinylated proteins by mass spectrometry. J. Proteome Res..

[125] Scheurer S.B., Roesli C., Neri D., Elia G. (2005). A comparison of different biotinylation reagents, tryptic digestion procedures, and mass spectrometric techniques for 2-d peptide mapping of membrane proteins. Proteomics.

[126] Elia G. (2012). Cell surface protein biotinylation for sds-page analysis. Methods Mol. Biol..

[127] Elia G. (2008). Biotinylation reagents for the study of cell surface proteins. Proteomics.

[128] Gyorgy P., Rose C.S., Eakin R.E., Snell E.E., Williams R.J. (1941). Egg-white injury as the result of nonabsorption or inactivation of biotin. Science.

[129] Chen B., Zhang A., Xu Z., Li R., Chen H., Jin M. (2011). Large-scale identification of bacteria-host crosstalk by affinity chromatography: Capturing the interactions of streptococcus suis proteins with host cells. J. Proteome Res..

[130] Yalow R.S., Berson S.A. (1960). Immunoassay of endogenous plasma insulin in man. J. Clin. Investig..

[131] Liedberg B., Nylander C., Lundstrom I. (1995). Biosensing with surface plasmon resonance—How it all started. Biosens. Bioelectron..

[132] Jerabek-Willemsen M., Wienken C.J., Braun D., Baaske P., Duhr S. (2011). Molecular interaction studies using microscale thermophoresis. Assay Drug Dev. Technol..

[133] Nesbitt S.A., Horton M.A. (1992). A nonradioactive biochemical characterization of membrane proteins using enhanced chemiluminescence. Anal. Biochem..

[134] Ornberg R.L., Harper T.F., Liu H. (2005). Western blot analysis with quantum dot fluorescence technology: A sensitive and quantitative method for multiplexed proteomics. Nat. Meth..

[135] Burnett T.A., Dinkla K., Rohde M., Chhatwal G.S., Uphoff C., Srivastava M., Cordwell S.J., Geary S., Liao X., Minion F.C. (2006). P159 is a proteolytically processed, surface adhesin of mycoplasma hyopneumoniae: Defined domains of p159 bind heparin and promote adherence to eukaryote cells. Mol. Microbiol..

[136] Bogema D.R., Deutscher A.T., Woolley L.K., Seymour L.M., Raymond B.B., Tacchi J.L., Padula M.P., Dixon N.E., Minion F.C., Jenkins C. (2012). Characterization of cleavage events in the multifunctional cilium adhesin mhp684 (p146) reveals a mechanism by which mycoplasma hyopneumoniae regulates surface topography. MBio.

[137] Deutscher A.T., Jenkins C., Minion F.C., Seymour L.M., Padula M.P., Dixon N.E., Walker M.J., Djordjevic S.P. (2010). Repeat regions r1 and r2 in the p97 paralogue mhp271 of mycoplasma hyopneumoniae bind heparin, fibronectin and porcine cilia. Mol. Microbiol..

[138] Deutscher A.T., Tacchi J.L., Minion F.C., Padula M.P., Crossett B., Bogema D.R., Jenkins C., Kuit T.A., Walker M.J., Djordjevic S.P. (2012). Mycoplasma hyopneumoniae surface proteins mhp385 and mhp384 bind host cilia and glycosaminoglycans and are endoproteolytically processed by proteases that recognize different cleavage motifs. J. Proteome Res..

[139] Seymour L.M., Deutscher A.T., Jenkins C., Kuit T.A., Falconer L., Minion F.C., Crossett B., Padula M., Dixon N.E., Djordjevic S.P. (2010). A processed multidomain mycoplasma hyopneumoniae adhesin binds fibronectin, plasminogen, and swine respiratory cilia. J. Biol. Chem..

[140] Seymour L.M., Falconer L., Deutscher A.T., Minion F.C., Padula M.P., Dixon N.E., Djordjevic S.P., Walker M.J. (2011). Mhp107 is a member of the multifunctional adhesin family of mycoplasma hyopneumoniae. J. Biol. Chem..

[141] Seymour L.M., Jenkins C., Deutscher A.T., Raymond B.B., Padula M.P., Tacchi J.L., Bogema D.R., Eamens G.J., Woolley L.K., Dixon N.E. (2012). Mhp182 (p102) binds fibronectin and contributes to the recruitment of plasmin(ogen) to the mycoplasma hyopneumoniae cell surface. Cell. Microbiol..

[142] Chen B., Peng Y., Valeja S.G., Xiu L., Alpert A.J., Ge Y. (2016). Online hydrophobic interaction chromatography-mass spectrometry for top-down proteomics. Anal. Chem..

[143] Tran J.C., Doucette A.A. (2009). Multiplexed size separation of intact proteins in solution phase for mass spectrometry. Anal. Chem..

[144] Kim K.H., Compton P.D., Tran J.C., Kelleher N.L. (2015). Online matrix removal platform for coupling gel-based separations to whole protein electrospray ionization mass spectrometry. J. Proteome Res..

[145] Vellaichamy A., Tran J.C., Catherman A.D., Lee J.E., Kellie J.F., Sweet S.M., Zamdborg L., Thomas P.M., Ahlf D.R., Durbin K.R. (2010). Size-sorting combined with improved nanocapillary liquid chromatography-mass spectrometry for identification of intact proteins up to 80 kda. Anal. Chem..

[146] Jorgenson J.W., Lukacs K.D. (1983). Capillary zone electrophoresis. Science.

[147] Li Y., Champion M.M., Sun L., Champion P.A., Wojcik R., Dovichi N.J. (2012). Capillary zone electrophoresis-electrospray ionization-tandem mass spectrometry as an alternative proteomics platform to ultraperformance liquid chromatography-electrospray ionization-tandem mass spectrometry for samples of intermediate complexity. Anal. Chem..

[148] Nesbitt C.A., Zhang H., Yeung K.K. (2008). Recent applications of capillary electrophoresis-mass spectrometry (ce-ms): Ce performing functions beyond separation. Anal. Chim. Acta.

[149] Monton M.R., Terabe S. (2004). Field-enhanced sample injection for high-sensitivity analysis of peptides and proteins in capillary electrophoresis-mass spectrometry. J. Chromatogr. A.

[150] Armenta J.M., Gu B., Thulin C.D., Lee M.L. (2007). Coupled affinity-hydrophobic monolithic column for on-line removal of immunoglobulin g, preconcentration of low abundance proteins and separation by capillary zone electrophoresis. J. Chromatogr. A.

[151] Wojcik R., Li Y., Maccoss M.J., Dovichi N.J. (2012). Capillary electrophoresis with orbitrap-velos mass spectrometry detection. Talanta.

[152] Han X., Wang Y., Aslanian A., Bern M., Lavallee-Adam M., Yates J.R. (2014). Sheathless capillary electrophoresis-tandem mass spectrometry for top-down characterization of pyrococcus furiosus proteins on a proteome scale. Anal. Chem..

[153] Sun L., Knierman M.D., Zhu G., Dovichi N.J. (2013). Fast top-down intact protein characterization with capillary zone electrophoresis-electrospray ionization tandem mass spectrometry. Anal. Chem..

[154] Wojcik R., Zhu G., Zhang Z., Yan X., Zhao Y., Sun L., Champion M.M., Dovichi N.J. (2016). Capillary zone electrophoresis as a tool for bottom-up protein analysis. Bioanalysis.

[155] Moini M. (2007). Simplifying ce-ms operation. 2. Interfacing low-flow separation techniques to mass spectrometry using a porous tip. Anal. Chem..

[156] Nemes P., Rubakhin S.S., Aerts J.T., Sweedler J.V. (2013). Qualitative and quantitative metabolomic investigation of single neurons by capillary electrophoresis electrospray ionization mass spectrometry. Nat. Protoc..

[157] Li Y., Compton P.D., Tran J.C., Ntai I., Kelleher N.L. (2014). Optimizing capillary electrophoresis for top-down proteomics of 30–80 kda proteins. Proteomics.

[158] Zhao Y., Sun L., Zhu G., Dovichi N.J. (2016). Coupling capillary zone electrophoresis to a q exactive hf mass spectrometer for top-down proteomics: 580 proteoform identifications from yeast. J. Proteome Res..

[159] Parks B.A., Jiang L., Thomas P.M., Wenger C.D., Roth M.J., Boyne M.T., Burke P.V., Kwast K.E., Kelleher N.L. (2007). Top-down proteomics on a chromatographic time scale using linear ion trap fourier transform hybrid mass spectrometers. Anal. Chem..

[160] Han X., Wang Y., Aslanian A., Fonslow B., Graczyk B., Davis T.N., Yates J.R. (2014). In-line separation by capillary electrophoresis prior to analysis by top-down mass spectrometry enables sensitive characterization of protein complexes. J. Proteome Res..

[161] Kelleher N.L., Senko M.W., Siegel M.M., McLafferty F.W. (1997). Unit resolution mass spectra of 112 kda molecules with 3 da accuracy. J. Am. Soc. Mass Spectrom..

[162] Loo J.A., Quinn J.P., Ryu S.I., Henry K.D., Senko M.W., McLafferty F.W. (1992). High-resolution tandem mass spectrometry of large biomolecules. Proc. Natl. Acad. Sci. USA.

[163] Henry K.D., McLafferty F.W. (1990). Electrospray ionization with fourier-transform mass spectrometry. Charge state assignment from resolved isotopic peaks. Org. Mass Spectrom..

[164] Gordon E.F., Mansoori B.A., Carroll C.F., Muddiman D.C. (1999). Hydropathic influences on the quantification of equine heart cytochromec using relative ion abundance measurements by electrospray ionization fourier transform ion cyclotron resonance mass spectrometry. J. Mass Spectrom..

[165] Zubarev R.A., Makarov A. (2013). Orbitrap mass spectrometry. Anal. Chem..

[166] Hu Q., Noll R.J., Li H., Makarov A., Hardman M., Graham Cooks R. (2005). The orbitrap: A new mass spectrometer. J. Mass Spectrom..

[167] McAlister G.C., Berggren W.T., Griep-Raming J., Horning S., Makarov A., Phanstiel D., Stafford G., Swaney D.L., Syka J.E., Zabrouskov V. (2008). A proteomics grade electron transfer dissociation-enabled hybrid linear ion trap-orbitrap mass spectrometer. J. Proteome Res..

[168] Anderson L.C., DeHart C.J., Kaiser N.K., Fellers R.T., Smith D.F., Greer J.B., LeDuc R.D., Blakney G.T., Thomas P.M., Kelleher N.L. (2017). Identification and characterization of human proteoforms by top-down lc-21 tesla ft-icr mass spectrometry. J. Proteome Res..

[169] Data Analysis Software Page. http://www.topdownproteomics.org/resources/software.

[170] Campbell J.L., Le Blanc J.C. (2010). Targeted ion parking for the quantitation of biotherapeutic proteins: Concepts and preliminary data. J. Am. Soc. Mass Spectrom..

[171] Muddiman D.C., Cheng X., Udseth H.R., Smith R.D. (1996). Charge-state reduction with improved signal intensity of oligonucleotides in electrospray ionization mass spectrometry. J. Am. Soc. Mass Spectrom..

[172] Reid G.E., Shang H., Hogan J.M., Lee G.U., McLuckey S.A. (2002). Gas-phase concentration, purification, and identification of whole proteins from complex mixtures. J. Am. Chem. Soc..

[173] McLuckey S.A., Reid G.E., Wells J.M. (2002). Ion parking during ion/ion reactions in electrodynamic ion traps. Anal. Chem..

[174] Ryan C.M., Souda P., Bassilian S., Ujwal R., Zhang J., Abramson J., Ping P., Durazo A., Bowie J.U., Hasan S.S. (2010). Post-translational modifications of integral membrane proteins resolved by top-down fourier transform mass spectrometry with collisionally activated dissociation. Mol. Cell. Proteom..

[175] Palumbo A.M., Reid G.E. (2008). Evaluation of gas-phase rearrangement and competing fragmentation reactions on protein phosphorylation site assignment using collision induced dissociation-ms/ms and ms3. Anal. Chem..

[176] Ahlf D.R., Compton P.D., Tran J.C., Early B.P., Thomas P.M., Kelleher N.L. (2012). Evaluation of the compact high-field orbitrap for top-down proteomics of human cells. J. Proteome Res..

[177] Smith R.D., Paša-Tolić L., Lipton M.S., Jensen P.K., Anderson G.A., Shen Y., Conrads T.P., Udseth H.R., Harkewicz R., Belov M.E. (2001). Rapid quantitative measurements of proteomes by fourier transform ion cyclotron resonance mass spectrometry. Electrophoresis.

[178] Pesavento J.J., Mizzen C.A., Kelleher N.L. (2006). Quantitative analysis of modified proteins and their positional isomers by tandem mass spectrometry: Human histone h4. Anal. Chem..

[179] Garcia B.A., Mollah S., Ueberheide B.M., Busby S.A., Muratore T.L., Shabanowitz J., Hunt D.F. (2007). Chemical derivatization of histones for facilitated analysis by mass spectrometry. Nat. Protoc..

[180] Huang H., Lin S., Garcia B.A., Zhao Y. (2015). Quantitative proteomic analysis of histone modifications. Chem. Rev..

[181] Williams T.L., Callahan J.H., Monday S.R., Feng P.C., Musser S.M. (2004). Relative quantitation of intact proteins of bacterial cell extracts using coextracted proteins as internal standards. Anal. Chem..

[182] Ntai I., Toby T.K., LeDuc R.D., Kelleher N.L. (2016). A method for label-free, differential top-down proteomics. Methods Mol. Biol..

[183] Unwin R.D. (2010). Quantification of proteins by itraq. Methods Mol. Biol..

[184] Unwin R.D., Griffiths J.R., Whetton A.D. (2010). Simultaneous analysis of relative protein expression levels across multiple samples using itraq isobaric tags with 2d nano lc-ms/ms. Nat. Protoc..

[185] Thompson A., Schafer J., Kuhn K., Kienle S., Schwarz J., Schmidt G., Neumann T., Johnstone R., Mohammed A.K., Hamon C. (2003). Tandem mass tags: A novel quantification strategy for comparative analysis of complex protein mixtures by ms/ms. Anal. Chem..

[186] Hsu J.L., Huang S.Y., Chow N.H., Chen S.H. (2003). Stable-isotope dimethyl labeling for quantitative proteomics. Anal. Chem..

[187] Gygi S.P., Rist B., Gerber S.A., Turecek F., Gelb M.H., Aebersold R. (1999). Quantitative analysis of complex protein mixtures using isotope-coded affinity tags. Nat. Biotechnol..

[188] Hung C.W., Tholey A. (2012). Tandem mass tag protein labeling for top-down identification and quantification. Anal. Chem..

[189] Burkhart J.M., Vaudel M., Zahedi R.P., Martens L., Sickmann A. (2011). Itraq protein quantification: A quality-controlled workflow. Proteomics.

[190] Cologna S.M., Crutchfield C.A., Searle B.C., Blank P.S., Toth C.L., Ely A.M., Picache J.A., Backlund P.S., Wassif C.A., Porter F.D. (2015). An efficient approach to evaluate reporter ion behavior from maldi-ms/ms data for quantification studies using isobaric tags. J. Proteome Res..

[191] Gouw J.W., Krijgsveld J., Heck A.J. (2010). Quantitative proteomics by metabolic labeling of model organisms. Mol. Cell. Proteom..

[192] Ong S.E., Mann M. (2006). A practical recipe for stable isotope labeling by amino acids in cell culture (silac). Nat. Protoc..

[193] Ong S.E., Blagoev B., Kratchmarova I., Kristensen D.B., Steen H., Pandey A., Mann M. (2002). Stable isotope labeling by amino acids in cell culture, silac, as a simple and accurate approach to expression proteomics. Mol. Cell. Proteom..

[194] Waanders L.F., Hanke S., Mann M. (2007). Top-down quantitation and characterization of silac-labeled proteins. J. Am. Soc. Mass Spectrom..

[195] Collier T.S., Hawkridge A.M., Georgianna D.R., Payne G.A., Muddiman D.C. (2008). Top-down identification and quantification of stable isotope labeled proteins from aspergillus flavus using online nano-flow reversed-phase liquid chromatography coupled to a ltq-fticr mass spectrometer. Anal. Chem..

[196] Picard G., Lebert D., Louwagie M., Adrait A., Huillet C., Vandenesch F., Bruley C., Garin J., Jaquinod M., Brun V. (2012). Psaq standards for accurate ms-based quantification of proteins: From the concept to biomedical applications. J. Mass Spectrom..

[197] Brun V., Dupuis A., Adrait A., Marcellin M., Thomas D., Court M., Vandenesch F., Garin J. (2007). Isotope-labeled protein standards: Toward absolute quantitative proteomics. Mol. Cell. Proteom..

[198] Dupuis A., Hennekinne J.A., Garin J., Brun V. (2008). Protein standard absolute quantification (psaq) for improved investigation of staphylococcal food poisoning outbreaks. Proteomics.

[199] Kippen A.D., Cerini F., Vadas L., Stocklin R., Vu L., Offord R.E., Rose K. (1997). Development of an isotope dilution assay for precise determination of insulin, c-peptide, and proinsulin levels in non-diabetic and type ii diabetic individuals with comparison to immunoassay. J. Biol. Chem..

[200] Stocklin R., Vu L., Vadas L., Cerini F., Kippen A.D., Offord R.E., Rose K. (1997). A stable isotope dilution assay for the in vivo determination of insulin levels in humans by mass spectrometry. Diabetes.

